# Deciphering the olfactory repertoire of the tiger mosquito *Aedes albopictus*

**DOI:** 10.1186/s12864-017-4144-1

**Published:** 2017-10-11

**Authors:** Fabrizio Lombardo, Marco Salvemini, Carmine Fiorillo, Tony Nolan, Laurence J. Zwiebel, José M. Ribeiro, Bruno Arcà

**Affiliations:** 1 Department of Public Health and Infectious Diseases, Division of Parasitology, Sapienza University of Rome, Rome, Italy; 2 Department of Biology, University of Naples Federico II, Naples, Italy; 3 Department of Life Sciences, Imperial College London, London, UK; 4 Department of Biological Sciences, Vanderbilt University, Nashville, TN USA; 5 NIAID, Laboratory of Malaria and Vector Research, NIH, Rockville, 20852 MD USA

**Keywords:** Mosquito, *Aedes albopictus*, Olfaction, Antennae, Maxillary palps, mRNA-sequencing, Chemosensory genes

## Abstract

**Background:**

The Asian tiger mosquito *Aedes albopictus* is a highly invasive species and competent vector of several arboviruses (e.g. dengue, chikungunya, Zika) and parasites (e.g. dirofilaria) of public health importance. Compared to other mosquito species, *Ae. albopictus* females exhibit a generalist host seeking as well as a very aggressive biting behaviour that are responsible for its high degree of nuisance. Several complex mosquito behaviours such as host seeking, feeding, mating or oviposition rely on olfactory stimuli that target a range of sensory neurons localized mainly on specialized head appendages such as antennae, maxillary palps and the mouthparts.

**Results:**

With the aim to describe the *Ae. albopictus* olfactory repertoire we have used RNA-seq to reveal the transcriptome profiles of female antennae and maxillary palps. Male heads and whole female bodies were employed as reference for differential expression analysis. The relative transcript abundance within each tissue (TPM, transcripts per kilobase per million) and the pairwise differential abundance in the different tissues (fold change values and false discovery rates) were evaluated. Contigs upregulated in the antennae (620) and maxillary palps (268) were identified and relative GO and PFAM enrichment profiles analysed. Chemosensory genes were described: overall, 77 odorant binding proteins (OBP), 82 odorant receptors (OR), 60 ionotropic receptors (IR) and 30 gustatory receptors (GR) were identified by comparative genomics and transcriptomics. In addition, orthologs of genes expressed in the female/male maxillary palps and/or antennae and involved in thermosensation (e.g. pyrexia and arrestin1), mechanosensation (e.g. piezo and painless) and neuromodulation were classified.

**Conclusions:**

We provide here the first detailed transcriptome of the main *Ae. albopictus* sensory appendages, i.e. antennae and maxillary palps. A deeper knowledge of the olfactory repertoire of the tiger mosquito will help to better understand its biology and may pave the way to design new attractants/repellents.

**Electronic supplementary material:**

The online version of this article (10.1186/s12864-017-4144-1) contains supplementary material, which is available to authorized users.

## Background

The Asian tiger mosquito *Aedes albopictus* is an aggressive daytime-biting vector of several arboviruses pathogenic to humans (e.g. dengue, chikungunya, Zika). *Ae. albopictus* has been described as one of the 100 worst invasive species in the world (Global Invasive Species Database, http://www.issg.org/database/). Its impact on human health relies indeed on its rapid and aggressive worldwide spread from its native home range (South-East Asia), along with its ecological adaptability in different traits, including most importantly feeding behaviours, diapause, and vector competence [1–5].

Biological signals captured from the surrounding environment and sensed through olfaction and other chemosensory modalities play a central role in the modulation of mosquito behaviours such as host-seeking, feeding, mating, oviposition and reception of repellents [6]. Olfactory responses are initiated by activation of olfactory sensory neurons (OSNs) localized mainly on antennae, maxillary palps, mouthparts (consisting of the proboscis and labellum) and tarsi [6]. These sensory appendages may perceive extremely diverse extrinsic stimuli, such as volatile and non-volatile odours or pheromones, temperature, humidity, mild or noxious touch, gravity, etc., to activate a complex mix of mosquito perception pathways [7–9]. The sensing of chemical stimuli, i.e. chemosensation, relies on chemosensory neurons that are selectively activated by various volatile compounds, such as odorant molecules and pheromones [10]. The molecular components underlying peripheral olfactory signalling encompass a range of intracellular and extracellular contexts. The complexity of olfactory factors together with differential expression and/or abundance directly contributes to the modulation of specific behaviours across mosquito species. From a receptor-centric perspective, chemosensation in insects is largely mediated by diverse members of three gene families expressed primarily in OSNs that reside within specialized sensilla that populate olfactory appendages: odorant receptors (ORs), gustatory receptors (GRs) and ionotropic receptors (IRs) (reviewed in [6, 11]). Odorants and chemical compounds cross the cuticle through sensillar pores to reach the aqueous sensillar lymph, and are then postulated to be recognized and carried to their cognate specific receptors on OSN dendritic membranes by members of a diverse family of extracellular odorant binding proteins (OBPs) and pheromone binding proteins (PBPs) that are secreted by a network of accessory cells localized at the base of insect sensilla (reviewed in [12]). There are several distinct types of chemosensory sensilla which house the OSNs along other sensory neurons and their associated accessory cells that populate mosquito sensory appendages [13].

From a cellular and molecular perspective, several studies concerning the activation of olfactory transduction pathways in insects suggest that ORs function as heteromeric complexes that form ligand gated ion channels in association with the ubiquitous co-receptor ORco [14, 15], that may also in some circumstances utilize G-protein coupled second messenger pathways to confer OSN odour sensitivity [16]. Activation of OSNs by odorants triggers complex behavioural responses [17], and several evidences highlight the crucial role of ORs in accomplishing this process in mosquitoes [18, 19]. Membrane-bound insect GRs are phylogenetically related to ORs and are generally expressed in gustatory receptor neurons (GRNs) found in chemosensory sensilla distributed on the mouthparts, wing margins, genitalia, and tarsal segments of the legs [20–23]. In the malaria mosquito *Anopheles gambiae* a suite of three highly conserved GRs is expressed in a unique array of non-OR containing maxillary palp OSNs that respond to volatile CO_2_ [24]. Similarly, in the dengue and yellow fever mosquito *Aedes aegypti* a crucial role in host preference/seeking was shown for two of the three GRs as component of the CO_2_ receptor [25, 26]. Insect IRs are not related to ORs or GRs, but instead represent an ancient family derived from ionotropic glutamate receptors (iGluRs), a highly conserved family of ligand-gated ion channels involved in neurotransmission as well as signalling mechanisms in response to external chemical stimuli in both eukaryotes and prokaryotes [27, 28]. Mosquito IRs are not as well characterized functionally as ORs, although recent studies in *An. gambiae* [29] are consistent with work from *Drosophila melanogaster* showing that IRs detect volatile compounds such as acids, ammonia, or amines [30]. Behavioural studies in *Ae. aegypti* showed that this mosquito uses polyamines both to find feeding sources and especially to locate egg-laying sites [31]: this attraction could be mediated by IRs, as demonstrated in the fruit fly [31]. In addition to the principal chemosensory receptor gene families, OSN membranes are also populated with several important families of olfactory genes. The sensory neuron membrane proteins (SNMPs), which belong to the scavenger receptor type B gene family (SCRB/CD36) [32, 33], are receptors involved in cell-cell communications, ligand (fatty acids) internalization [34] and pheromone detection as shown in *D. melanogaster* [35, 36]. Insect SNMPs are well-conserved and are typically expressed in neurons or support cells associated with sensilla [34].

Beyond those membrane components, a range of secreted proteins makes essential extracellular contributions to olfactory signalling pathways. Of these, OBPs are highly expressed water-soluble components of sensillary lymph that are hypothesized to bind and solubilize odorant compounds from the external environment and to transport them to their respective olfactory receptors triggering olfactory transduction pathways [12]. Insect OBPs are 10–30 kDa globular proteins characterized by six α-helical domains comprising of six highly conserved cysteines with specific disulphide connectivity. In mosquitoes three sub-families of OBPs have been characterized so far: (i) Classic OBPs, carrying the six conserved cysteines typical of the OBP family; (ii) PlusC OBPs, with the same conserved cysteines and disulphide connectivity but also containing six additional cysteines; (iii) Atypical OBPs, which are among the longest known OBPs including two domains that are homologous to the Classic OBP domain and are hence considered as “dimer OBPs” [37, 38]. Moreover, OBPs lacking C2 and C5 cysteines are commonly widespread among studied organisms and are named MinusC [38]. Structural studies in mosquito described the adaptability of OBP binding site to accommodate several ligands [39, 40]. It was also demonstrated that some OBP might show a binding preference for certain odour molecules. For instance, the presence of OBP1 in *An. gambiae* mediates the binding with the ligand indole [41], a known oviposition attractant for the southern house mosquito *Culex quinquefasciatus* [42]. OBPs therefore likely represent a critical initial interface between the environment and the mosquito: the variety of OBPs and chemosensory receptors each mosquito possesses and expresses at any one time is likely to influence specific behavioural features (i.e., food, host and oviposition site preference and seeking) as reflected in their capacity to sense diverse sets of attractants and repellents. In addition to OBPs, there are several other families of secreted proteins involved in peripheral chemosensation. Among these, members of *CheA* and *CheB* gene families, which encode small soluble proteins [43], were found expressed in sex-specific patterns and involved in the detection of cuticular hydrocarbons required for the courtship in the fruit fly [44]. CheB protein may interact with degenerin/epithelial Na^+^ channels (pickpocket, ppk) to detect these contact pheromones [43]. The molecular mechanism of CheA and CheB function remains unknown, but may involve interaction with other membrane bound receptors or ppk channels [43]. Another class of secreted extracellular proteins playing crucial roles in olfaction are the odorant/pheromone-degrading enzymes (ODEs/PDEs), which are involved in the degradation of odorants thus clearing sensillar lymph [45].

Sensory appendages such as tarsi and head appendages (labella, proboscis, maxillary palps and antennae) are not only involved in chemosensation but also play essential roles in mediating mosquito sensitivity to various additional stimuli that include temperature, dangerous and mild touch, humidity, gravity and other sensory modalities [6]. In this respect there are several families of membrane-bound molecules playing relevant roles. The pickpocket (ppk) and the transient receptor potential channels (trp) gene families have potential roles in insect taste, thermo- and mechano-reception [6, 46]. Indeed, mosquitoes not only must avoid extreme cold and heat but also accurately sense temperature as host-seeking females are known to be attracted to a narrow, specific temperature range associated with vertebrate hosts [6]. In *D. melanogaster*, GR28 has been involved in the detection of rapid temperature changes in adult flies [47]; in mosquitoes, *TrpA1*, *Painless*, *Pyrexia* and *PlcB* play roles in the detection of harmful heat thresholds and functional studies have directly implicated mosquito TRPA1 in thermo-sensitivity in both *An. gambiae* [48] and *Ae. aegypti* [49]. In addition, *Painless*, *Pyrexia* and *PlcB* appear also to be involved in light detection. Mechanosensation is the entire repertoire of actions and reactions related to the detection of sound, gravity and mild/noxious touch [46]. Previous studies in the fruit fly identified genes involved in some aspects of mechanosensation: for example the proteins painless, piezo and pickpocket (Ppk1) are mainly involved in perception of noxious touch [50–52], whereas nompC (no mechanoreceptor potential C), Ppk2, chloride channel-b, narrow abdomen, mrityu, the ionotropic receptors Nmdar1 and Nmdar2 are instead required for mild touch detection [53, 54].

The worldwide spread of the tiger mosquito *Ae. albopictus*, its competence in the transmission of several pathogens and the recent involvement in arboviral outbreaks [55–60], point out the need to acquire a deeper knowledge on crucial aspects of its life cycle that may help developing novel and more effective strategies for its control [9]. Here we employ an RNA-seq based approach to generate a comprehensive transcriptome of *Ae. albopictus* female antennae and maxillary palps. This facilitates the characterization of the main *Ae. albopictus* gene families involved in the perception of olfactory stimuli as well as of several additional transcripts likely implicated in sensing taste, temperature, humidity, touch, injury and gravity. We believe that the assembly of this olfactory repertoire will be useful for improving the annotation of the *Ae. albopictus* genome [61, 62]. Moreover, the comparison to other mosquito species may help understanding some particular behavioural and ecological features of the tiger mosquito (niche adaptation, invasiveness, feeding habits) that in the long run it may contribute to the development of novel control strategies.

## Methods

### Insects and tissue dissections

The *Aedes albopictus* strain used in this study was originally collected in Rome in 2012 and reared in the insectary for several generations (eggs kindly provided by Roberto Romi and Marco Di Luca, Istituto Superiore di Sanità, Rome, Italy). Mosquitoes were reared under standard laboratory conditions (25 ± 1 °C, relative humidity 60 ± 10%, light:dark photoperiod 14:10 h) in the insectary of the Department of Public Health and Infectious Diseases at Sapienza University, Rome. Adult females (2–6 days post-emergence, dpe) maintained on a 10% sucrose diet were used in this study. The age range was selected in the attempt to reveal transcripts encoding most of the olfactory factors involved in the preference, selection and location of blood-meal hosts in female mosquitoes that play critical roles in significantly establishing their vectorial capacity. Indeed, it is known that mosquitoes do not seek blood source in the first 24–48 h post-emergence [63], and temporal expression analyses showed that the largest transcriptional increase of olfactory genes occurs up to day 4 dpe in *An. gambiae* [64] and up to day 6 in *Ae. aegypti*, followed by a plateau in 10 dpe mosquitoes [65]. At 2 to 4 h after the gradual onset of light in rearing chambers (“insectary sunrise”) which corresponds to Zeitgeber time (ZT) 2 to ZT4, mosquitoes were anaesthetized using ice (2–3 min), and female antennae, female palps, male heads (with all the appendages attached) were hand-dissected, frozen in liquid nitrogen, and stored at −80 °C until needed. Whole females were directly frozen and stored as above. Two independent collections of 500 female antennae, 500 female palps, 10 adult females and 15 male heads were used for RNA extractions to obtain two independent biological replicates.

### RNA extraction, library preparation and sequencing

RNA was extracted using Trizol® Reagent (Life Technologies) according to the manufacturer’s protocol. Dissected tissues and entire females were initially crushed with pestles in Trizol reagent before proceeding with the extraction protocol. RNA concentration and quality were evaluated using standard procedures: Take3 Module (BioTek SynergyHT) reading and gel electrophoresis. RNA samples were treated with DNAseI (Ambion). Amounts of total RNA used to synthesize mRNA TruSeq libraries were the following: 500 Antennae (A): 1.4 μg; 500 Antennae (B): 1.9 μg; 500 Palps (A): 500 ng; 500 Palps (B): 400 ng 10 Female Body (A): 4.5 μg; 10 Female Body (B): 4.2 μg; 15 Male Heads (A): 3.3 μg; 15 Male Heads (B): 3 μg. cDNA library preparation, including fragmentation and barcoding (ligation of specific adapters), was performed following the Illumina TruSeq RNA Library v2 protocol (Illumina, San Diego, CA, USA). After quality evaluation with an Agilent 2010 Bioanalyzer, as recommended for Illumina sequencing, the libraries were diluted with an elution buffer and loaded on an Illumina HiSeq2000 for sequencing (paired-end, PE, 2 × 100 bp), and each cDNA library was sequenced.

### Assembly, sequence annotation and expression profiling

Clean reads were generated from raw reads by removing adaptor sequences, ambiguous reads, and low-quality reads with a qual value cutoff of 15. De novo assembly of clean data was accomplished by ABySS® and SOAPdenovo-trans® software, using several k-mers (every 10th from 25 to 95). Quality control between RNA-seq replicates was performed using the PtR Trinity perl script (release 2014–07-17) prior to merging of the duplicate (A/B) assemblies. Merged assembly (BLAST and cap3 assembler) was used to extract putative protein coding sequences (CDS). When the *Ae. albopictus* genome was made available [61, 62], we have compared (by BLAST) the VectorBase AaloF1 gene annotation [62] with the olfactory transcriptome predictions to assign VectorBase ID and reduce redundancy (annotation Software @CBS-DTU). Coding sequences (CDS) were extracted based on the existence of a signal peptide in the longer open reading frame (ORF) and by similarities to other proteins found in the Refseq invertebrate database from the National Center for Biotechnology Information (NCBI), proteins from Diptera deposited at NCBI’s Genbank and from SwissProt. To identify gene categories and PFAM terms enriched in the pairwise comparisons between the four tissues a GO/PFAM term enrichment analysis was performed using the Annocript software [66] and the Fisher Exact Test (adjusted *p*-value <0.01) in R package [67]. Clean reads were mapped back onto the assembled transcriptome using RSEM software and read count for each gene was obtained from the mapping results. Expression levels were assessed in terms of TPM values (transcripts per kilobase per million reads), which were calculated based on the number of mapped transcript fragments corrected for transcript length and sequencing depth. Differential expression analysis of two samples was performed using the edgeR R package [68]. *P* value was adjusted using FDR (False Discovery Rate). Then, paired comparisons were conducted in the following manner: female antennae vs. female body, female palps vs. female body and male heads vs. female body. EdgeR was run only for transcripts having at least one library with RPKM equal or larger than one, and only contigs with 2 < logFC < −2 were considered. Pairwise comparisons using edgeR were done using the generalized linear model (GLM) likelihood ratio test.

### Identification of chemosensory genes, sequence alignment and phylogenetic analysis

As mentioned in the paragraph above, each contig was analysed by BLAST analysis interrogating several databases. Contigs showing significant matches with proteins involved in chemosensation in different insects (the mosquitoes *An. gambiae*, *C. quinquefasciatus* and *Ae. aegypti* and other insects such as the fruit fly *D. melanogaster*) were identified. Moreover, a reciprocal BLAST analysis was carried out using FASTA sequences of *Ae. aegypti* OR [69–71], GR [22, 69, 71], IR [27, 69, 71], OBP [38, 69, 71] and of genes involved in more general sensorial functions [71]. True orthologs were therefore identified by reciprocal best-hit BLASTP analysis screening the *Ae. albopictus* olfactory transcriptome. These lists were then manually refined by TBLASTN searches taking also into account other relevant biological features such as high percentages of identity over shorter regions or possible gene duplication events. Deduced protein sequences were aligned using Clustal Omega on line tool at EMBL-EBI (http://www.ebi.ac.uk/Tools/msa/clustalo/). Alignments reported in this manuscript should be considered as provisional and susceptible of improvement, especially considering the limited overall reliability of the present genome annotation. Nevertheless, they provide clear clues and justification for the classification in the different families and subfamilies. Phylograms were obtained by using MEGA 5.0 software and Maximum likelihood protocol. Bootstrapping was performed by the re-sampling amino acid positions of 1000 replicates (Additional file 1).

### Quantitative real-time PCR

To verify transcript abundance patterns from RNA-seq analysis, RT-qPCR reactions were performed on eleven selected genes. Two novel batches of both antennae and maxillary palps were dissected from *Ae. albopictus* females 2–5 dpe (days post emergence); two batches of whole female mosquitoes were also collected. Total RNA was extracted using Trizol® reagent (Invitrogen) following manufacturer’s instruction and finally resuspended in DNAse-RNAse-free ultrapure ddH_2_O. Quantity and quality of RNA was evaluated by spectrophotometric measurement (using the Take3 module of plate reader BioTek SynergyHT and GEN5™ software) and agarose gel electrophoresis, respectively. DNAseI (using Ambion DNA-free kit®, per manufacturer’s instruction) treatment was followed by standard PCR (without Reverse Transcription) to verify efficacy of treatment. DNAseI-treated total RNA (~ 1 μg for dissected tissues and 5 μg for whole females) was used as substrate to generate First-Strand cDNA using SuperScript II RT (Invitrogen) and OligodT (Invitrogen). The synthesized cDNA samples were diluted to 5 ng/μl: in each qPCR reaction 2 μl of cDNA were used. For each gene, a standard curve was included in each plate, together with the endogenous reference gene (ribosomal protein S5, RpS5, AALF013336, [72, 73]). cDNA templates for the standard curve were obtained from whole female RNA samples, a dilution series starting from 100 ng/reaction was followed by 1:5 dilutions. cDNA templates were mixed with 2× PowerUp™ SYBR™ Green Master Mix (Applied Biosystem) and specific primers as listed in Additional file 2: Table S6. Each reaction included an initial holding stage of 2 min at 50 °C and of 2 min at 95 °C, followed by 40 cycles of PCR (95 °C, 15 s; 60 °C, 1 min); a final stage to obtain melting curves was also included in each plate, with detection steps every 0.3 °C. In relative quantification, the relative amount of each gene in each tissue is determined by the ratio between the amounts of target gene and endogenous reference gene calculated using their Ct values and the corresponding standard curves. This ratio is then compared between different samples, choosing a sample as calibrator. The relative transcription levels normalized by reference gene were compared with expression levels of RNA-seq. We initially evaluated the correlation between duplicates in both RNA-seq and qPCR datasets. To obtain values suitable for statistical comparisons, we calculated (for each gene) a fold-change (FC) value as the ratio of abundance over the group median. Duplicates were then averaged to obtain a single FC value for each gene. These values were used to evaluate the correlation between RNA-seq and qPCR methods, applying statistical evaluation throughout Spearman and Pearson tests. For both techniques, statistical evaluation (Spearman and Pearson tests) revealed also a significant linear correlation between duplicates (Additional file 3: Figure S11 A-D).

## Results and discussion

### *Transcriptome sequencing,* de novo *assembly and genome release*

**Fig. 1:**
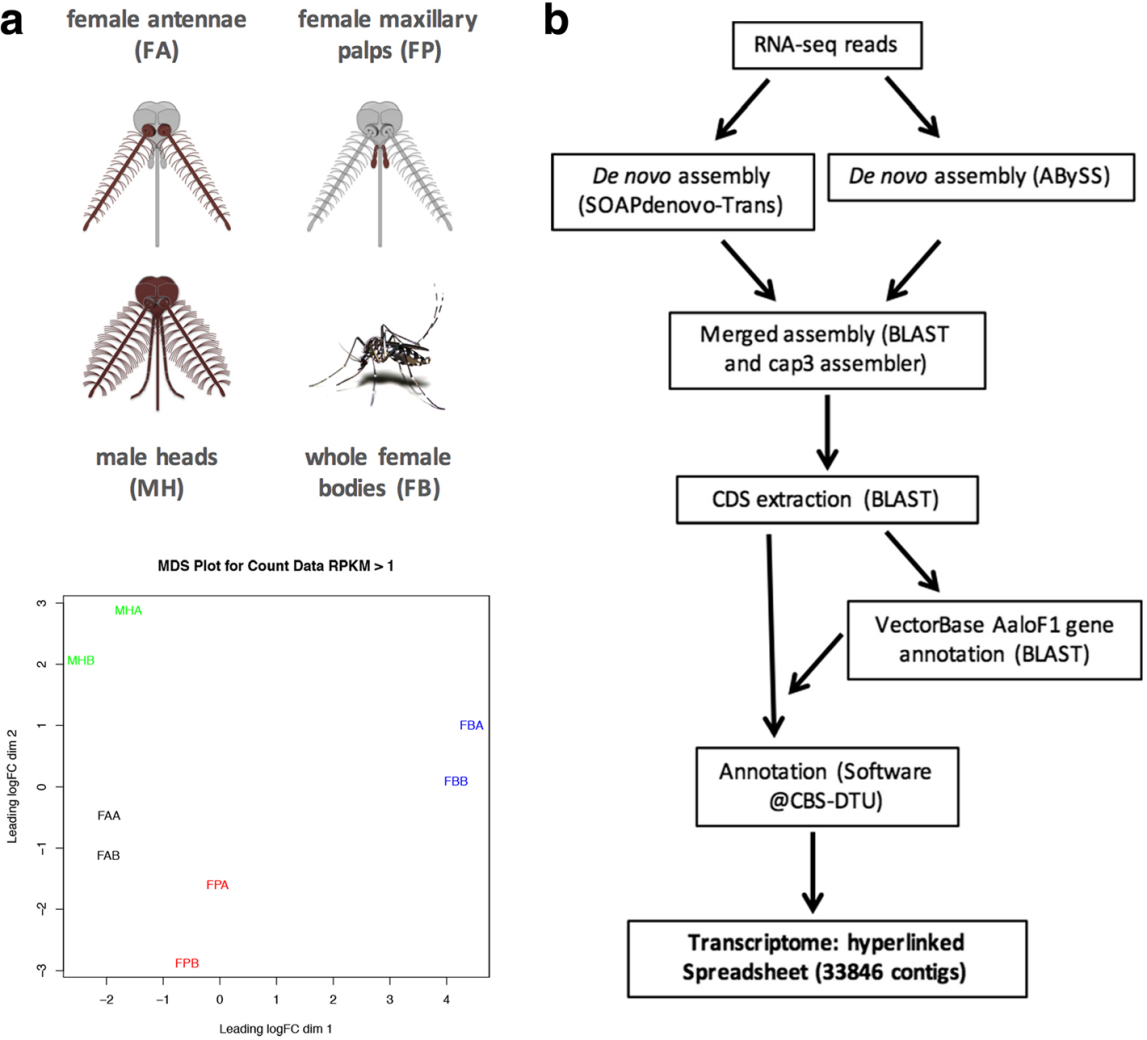
Tissues, RNA-seq method and cluster analysis. **a**. Schematic representation of female head with highlighted in red antennae and palps (upper part of the panel), male head and whole female mosquito (lower part of the panel). **b**. Workflow chart of procedures used for the assembly and the annotation of the *Ae. albopictus* olfactory transcriptome. **c**. Multidimensional plot of RNA-seq duplicates used in this study

Duplicate transcriptomes of olfactory appendages of the tiger mosquito *Ae. albopictus* were obtained for each of the four samples and general assembly statistics are summarized in Table 1 and Fig. 1. A comprehensive transcriptome was produced following de novo assembly procedures. Extracted CDS were compared by BLAST analysis to predicted peptides from the recently released *Ae. albopictus* genome [AaloF1 database, Foshan strain, [62]]. We found 6069 sequences that had at least 95% amino acid identity and at least 95% coverage to the Foshan predictions. Of these, 262 were at least 5% longer than the predictions. 20,085 contigs had less than 95% identity to the genomic predictions, and included 972 transcripts that produced >75% coverage and >50% identity to proteins from Diptera downloaded from GenBank. Altogether, we submitted 1842 protein coding sequences to the Transcriptome Shotgun Assembly (TSA) portal of the NCBI. This procedure produced a final spreadsheet of 33,846 contigs (Additional file 4: Dataset S1, Additional file 5: Dataset S2, Additional file 6: Dataset S3, Additional file 7: Dataset S4 and Fig. 1) and allowed assigning a VectorBase ID code (AALFxxxxxx) to 14,386 transcriptome contigs and significantly reducing redundancy. Mapping the reads to the transcriptome revealed an overall acceptable correlation between replicates for each tissue (Fig. 1c). The maxillary palp samples of females showed the highest divergence (but still highly correlated) whereas, as expected, reads derived from head tissues (female antennae, FA; female palps, FP and male heads, MH) displayed a higher correlation between them when compared to the female whole body (FB). Finally, a detailed cluster analysis was performed after assessment of TPM (transcripts per million) values for each replicate dataset (Additional file 8: Figure S1). This analysis confirmed the overall quality of replicates in our RNA-seq procedure and facilitated the identification of several groups of co-expressed genes that represent transcriptome profile signatures of the different tissues/sexes.

**Table 1:** Summary of sequencing and assembly statistics

Female Antennae A (reads)	29,945,374
Female Antennae B (reads)	17,712,800
Female Palps A (reads)	7,698,073
Female Palps B (reads)	5,354,526
Female Whole body A (reads)	25,639,847
Female Whole body B (reads)	19,011,547
Male Heads A (reads)	8,457,785
Male Heads B (reads)	19,688,382
Total bases (bp)	133,508,334
Total assembled bases (bp)	27,866,796
Assembled contigs	33,846
Average contig length (bp)	823
N50 (bp)	1563
Shortest transcript length (bp)	150
Longest transcript length (bp)	32,463
transcripts >1Kb	8953
transcripts >2Kb	3100

### Transcript abundance profiling in chemosensory tissues

**Fig. 2:**
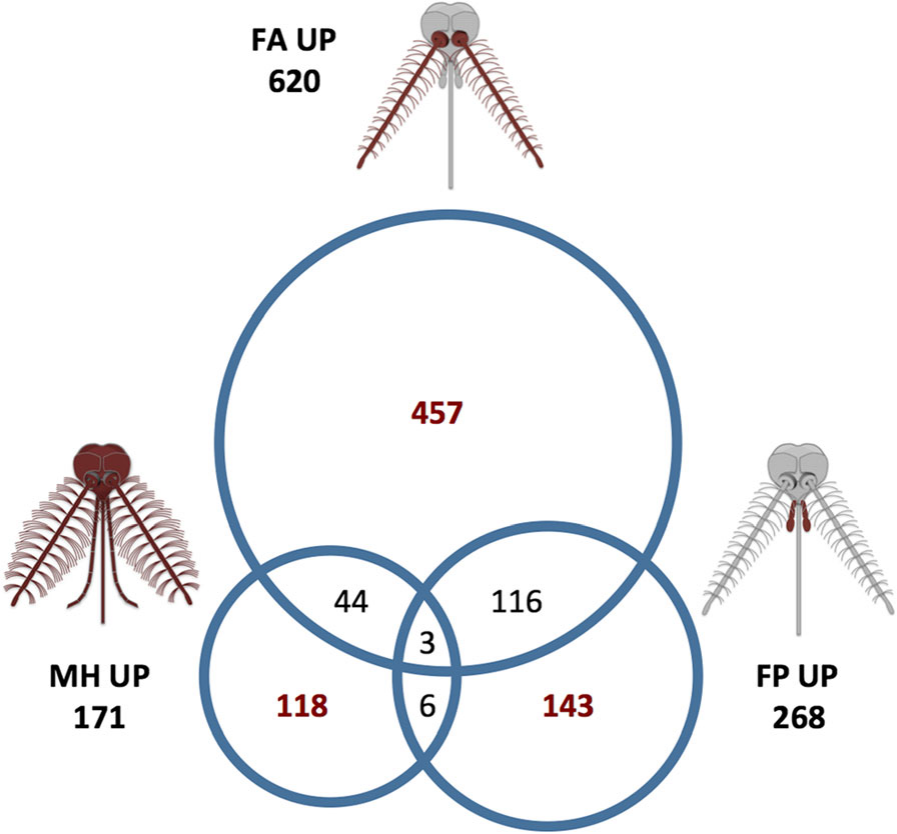
Pairwise sample comparisons. Proportional Venn diagram showing pairwise comparisons between female antennae, female palps and male heads. Gene subsets enhanced in each sample versus female body according to the edgeR threshold at *P* < 0.001 (see Table 2) were compared to each other. Overlaps (and relative numbers) represent the subsets of genes that are differentially expressed in more than one tissue

**Fig. 3:**
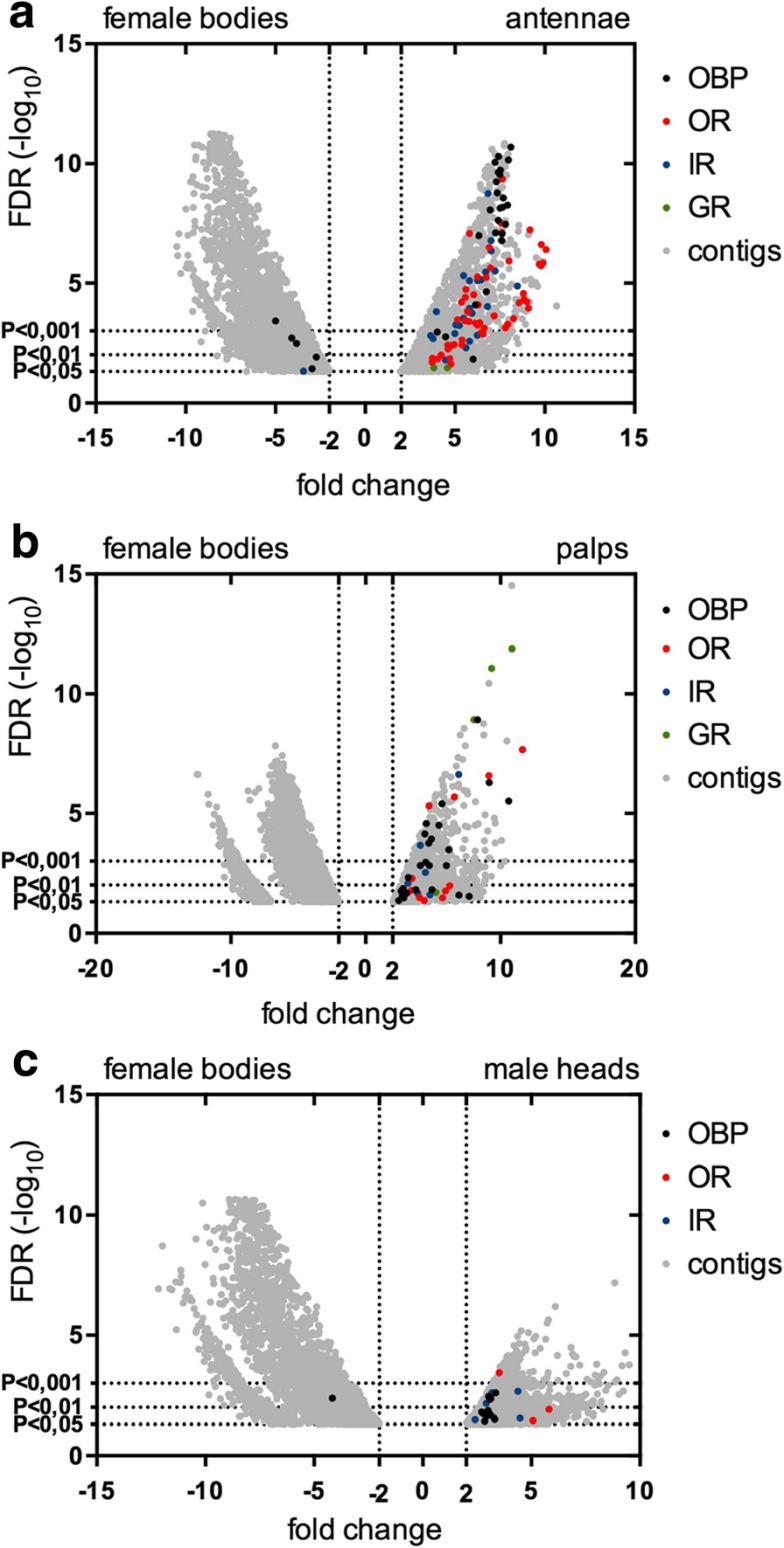
Differential expression (DE) of chemosensory genes. Volcano plots show the relative expression of contigs in pairwise comparisons. The x-axis represents the logFC (fold change) between tissues. The y-axis represents the negative log10 of the *p*-value (false discovery rate) as calculated by the Fisher’s Exact test. **a**. Female bodies vs Antennae. **b**. Female bodies vs Palps. **c**. Female bodies vs Male Heads. Only differentially expressed contigs (*P* < 0.05, logFC <−2 and >2) are shown in the plot (grey dots) with OBP indicated in black, OR in red, GR in green and IR in blue

Tissue-specific transcript enrichment was evaluated by pairwise comparisons between samples. Fold change (FC) Logarithmic values and false discovery rates (FDR) were calculated using edgeR to provide a statistical validation (Additional file 6: Dataset S3). We selected transcripts (represented hereafter as genes) showing enhanced abundance in chemosensory tissues (female antennae, female palps and male heads) when compared to female whole body and compared them to each other (Figs. 2 and 3). A strict statistical FDR threshold (*P* < 0.001) was used to define subsets of highly enhanced genes in female antennae (620 contigs) and in female palps (268 contigs, Table 2 and Figs. 2 and 3. Within these criteria, 171 contigs were enriched in male heads as compared to female body, indicating a sex-biased abundance profile (Fig. 3). Previous transcriptomic analysis in *An gambiae* and *Ae. aegypti* mosquitoes reported 2277 and 244 contigs differentially enhanced in female antennae, respectively [71, 74].

**Table 2:** Gene expression profiling by edgeR

		*P* < 0.05	*P* < 0.01	*P* < 0.001
FA vs FB	**FA UP:**	**1992**	**1129**	**620**
	FB UP:	3068	1962	1207
	Total:	5060	3091	1827
FP vs FB	**FP UP**	**1752**	**731**	**268**
	FB UP	3445	1755	807
	Total:	5197	2486	1075
MH vs FB	**MH UP**	**2137**	**819**	**171**
	FB UP	2918	1768	1091
	Total:	5055	2587	1262

Table 2. Gene expression profiling by edgeR. Pairwise comparisons (FA vs FB, FP vs FB and MH vs FB) highlight genes upregulated in each sample according to different probability thresholds (P < 0.05, *P* < 0.01 and P < 0.001)

Evaluation of both PFAM and GO enrichment in the enhanced gene sets was performed (Tables 3-5 and Additional file 9: Figure S2). The PFAM analysis was performed selecting the gene sets identified by the restrictive FDR < 0.001 threshold to increase the likelihood of isolating “tissue-specific” contigs. Not surprisingly, the three most frequent gene families in the antennae-enriched subset are 7tm Odorant receptor, PBP/GOBP and Ligand-gated ion channel (Table 3), similarly to previous observations in *An. gambiae* [74]. For the maxillary palps, the most significantly enhanced gene family comprises the ninjurin genes that are involved in several biological processes such as cell-cell, cell-matrix adhesion and nervous tissues development (Table 4). Other gene families enriched in the *Ae. albopictus* maxillary palps are the 7tm Chemosensory receptor (gustatory receptors), PBP/GOBP family and CD36 family that were also shown enriched in the maxillary palps of *An. gambiae* mosquitoes [74]. Finally, enriched gene families in male heads are represented mainly by Neurotransmitter-gated ion-channel ligand binding domain and 7 transmembrane receptor (rhodopsin family), since composed eyes and ganglia are large and abundant organs in this body part (Table 5).

**Table 3:** PFAM enrichment in *Ae. albopictus* female antennae vs transcriptome

PFAM TERM	PFAM DESCRIPTION	N° PFAM Transcriptome	N° PFAM FA UP 620	*p* value	FDR
PF02949	7tm Odorant receptor	77	35	3.74E-116	1.62E-113
PF01395	PBP/GOBP family	111	23	2.56E-38	5.54E-36
PF00060	Ligand-gated ion channel	45	14	1.50E-33	2.16E-31
PF03028	Dynein heavy chain and region D6 of dynein motor	16	6	5.12E-16	5.54E-14
PF03148	Tektin family	9	4	1.48E-11	1.28E-09
PF00025	ADP ribosylation factor	17	4	3.92E-07	1.70E-05
PF04923	Ninjurin	17	4	3.92E-07	1.70E-05
PF13414	TPR repeat	29	5	6.95E-07	2.74E-05
PF00043	Glutathione S-transferase. C-terminal domain	12	3	1.81E-05	0.00052
PF00250	Forkhead domain	18	3	0.00057	0.01179
PF04415	Protein of unknown function (DUF515)	19	3	0.00084	0.01671
PF02497	Arterivirus glycoprotein	7	2	0.00089	0.01692
PF03392	Insect pheromone-binding family. A10/OS-D	20	3	0.00121	0.02198

Table 3. PFAM enrichment in *Ae. albopictus* female antennae vs transcriptome. Summary of Protein families statistically overrepresented in tissue-enriched subset, FA-UP

**Table 4:** PFAM enrichment in *Ae. albopictus* female maxillary palps vs transcriptome

PFAM TERM	PFAM DESCRIPTION	N° PFAM Transcriptome	N° PFAM FP UP 268	p value	FDR
PF04923	Ninjurin	17	4	2.67E-16	1.32E-14
PF08395	7tm Chemosensory receptor	33	5	1.76E-14	6.96E-13
PF01395	PBP/GOBP family	111	8	1.27E-11	4.19E-10
PF01130	CD36 family	17	3	3.81E-09	1.08E-07
PF00650	CRAL/TRIO domain	40	4	8.93E-08	2.21E-06
PF06585	Haemolymph juvenile hormone binding protein (JHBP)	26	3	2.10E-06	3.77E-05
PF00188	Cysteine-rich secretory protein family	29	3	7.86E-06	0.00012
PF00106	short chain dehydrogenase	71	4	0.00015	0.00095
PF02958	Ecdysteroid kinase	51	3	0.00150	0.00663
PF00135	Carboxylesterase family	66	3	0.00815	0.02086
PF02949	7tm Odorant receptor	77	3	0.01891	0.04150

Table 4**.** PFAM enrichment in *Ae. albopictus* female maxillary palps vs transcriptome**.** Summary of Protein families statistically overrepresented in tissue-enriched subset, FP-UP

**Table 5:** PFAM enrichment in *Ae. albopictus* male heads vs transcriptome

PFAM TERM	PFAM DESCRIPTION	N° PFAM Transcriptome	N° PFAM MH UP 171	p value	FDR
PF02931	Neurotransmitter-gated ion-channel ligand binding domain	29	3	5.56E-09	2.06E-07
PF00001	7 transmembrane receptor (rhodopsin family)	81	4	2.60E-06	1.48E-05
PF00400	WD domain. G-beta repeat	86	3	0.00216	0.00533

Table 5**.** PFAM enrichment in *Ae. albopictus* male heads vs transcriptome**.** Summary of Protein families statistically overrepresented in tissue-enriched subset, MH-UP

Enrichment analysis of GO terms was performed comparing GO terms frequencies in tissue-specific subsets (*P* < 0.01) with GO terms frequencies in the transcriptome. Statistically significant GO terms (cellular component, molecular function and biological process) are shown in Additional file 9: Figure S2. By this analysis, OBPs (in both antennae-UP and palps-UP specific subsets) as well as odorant and ionotropic glutamate receptors (in the antennae-UP subset) are among the most highly represented molecular functions. Structural features such as dynein complex (cellular component) and microtubule-based movement and cilium assembly (biological process) are also significantly represented among antennae-specific GO terms.

*Ae. albopictus* antennae and palps are not only distinguishable as a result of significant differences in both PFAM and GO enrichment analyses but also when looking at specific and shared transcripts (i.e., contigs specific to the antennae, to the palps and to male heads as well as those whose expression was enhanced in more than one chemosensory appendage). These relationships are represented in a proportional Venn diagram (Fig. 2) that highlights the overlaps between tissues (antennae and palps) as well as tissue- and sex-specific groups. Comparison between the subset of female antennae-enriched contigs with those enriched in palps revealed a relevant overlap, with 119 enriched transcripts (44% of palp set) in common (Fig. 2). Around 28% of transcripts enhanced in male heads are also enhanced in the female antennae, while only 5% of palps-specific contigs are enhanced in male head. This comparison also identified groups of transcripts specific to the female antennae (457/620, 73%), female palps (143/268, 53%) and male heads (118/171, 69%).

### Chemosensory gene families

**Fig. 4:**
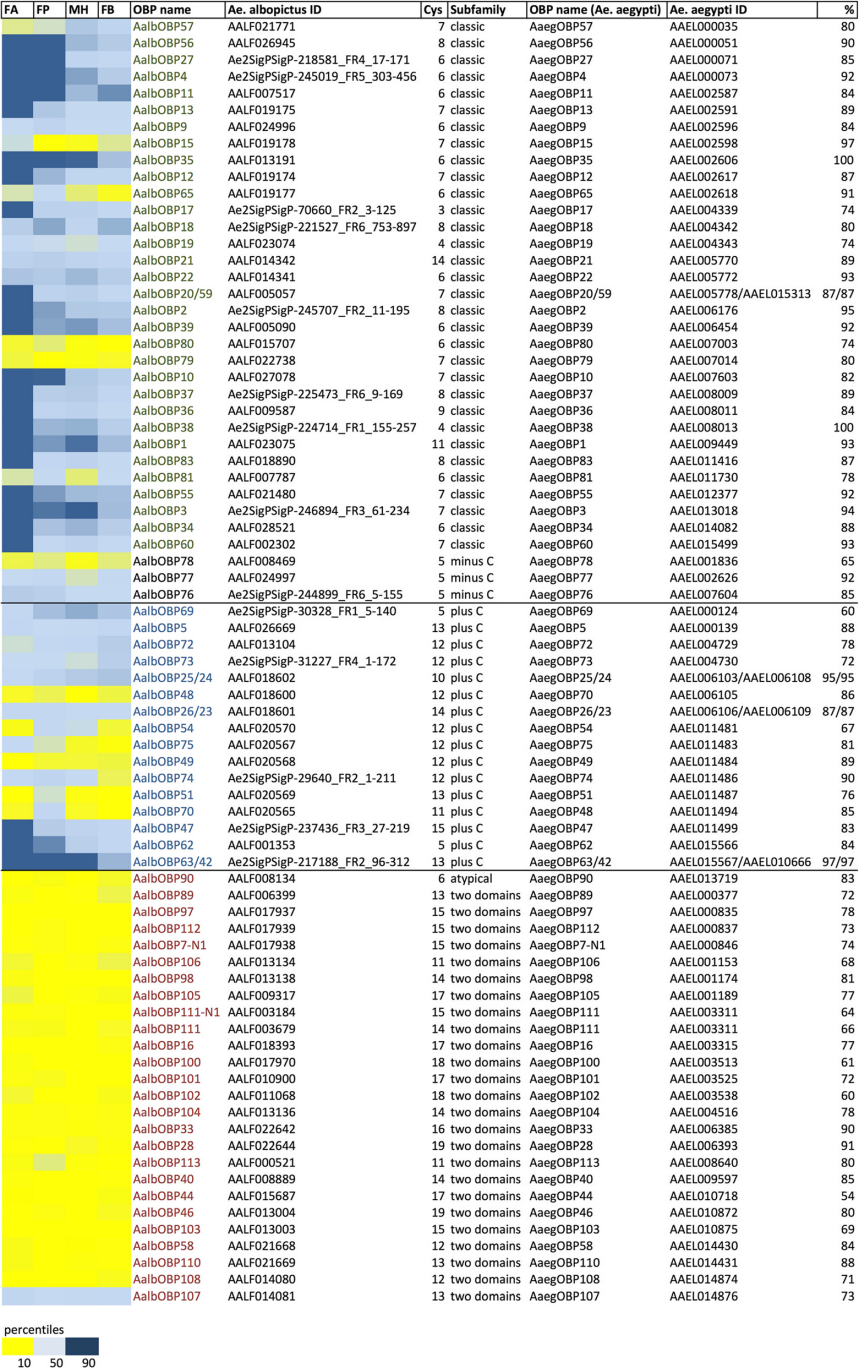
Odorant binding proteins (OBPs) in *Ae. albopictus* transcriptome. Left panel, abundance profile map: intensity scale (color gradient from blue to yellow indicates levels from high TPM to low) as indicated at the bottom. FA, female antennae; FP, female palps; MH male heads and FB, female body. Assigned OBP names, ID in *Ae. albopictus* (VectorBase ID AALFxxxxxx when available or contig ID in the transcriptome), number of cysteines, OBP subfamily, name and ID of the presumed *Ae. aegypti* ortholog and percentage of identity are reported

**Fig. 5:**
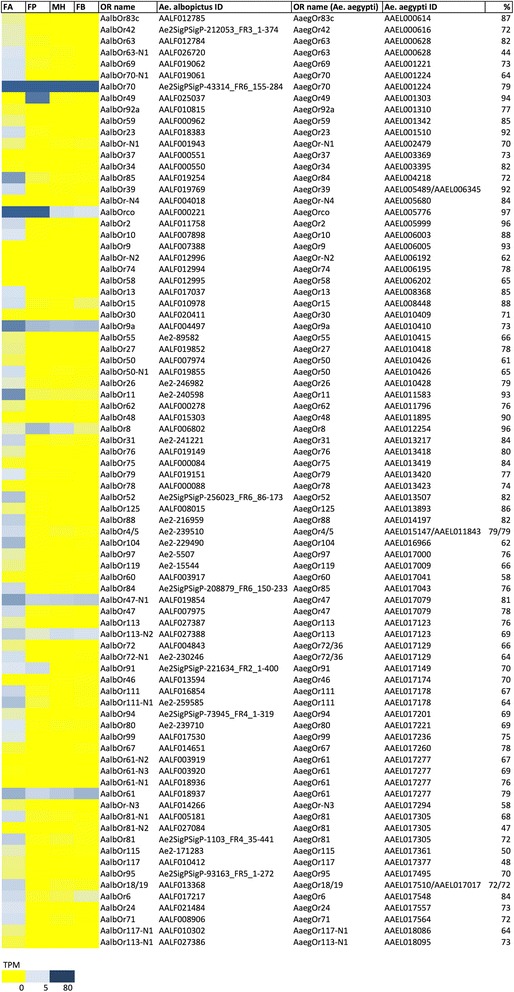
Odorant receptors (OR) in *Ae. albopictus* transcriptome. Left panel, abundance profile map: intensity scale (color gradient from blue to yellow indicates levels from high TPM to low) as indicated at the bottom. FA, female antennae; FP, female palps; MH male heads and FB, female body. OR name and *Ae. albopictus* ID columns indicate respectively the name assigned to OR in *Ae. albopictus* and the ID (VectorBase ID AALFxxxxxx when available or contig ID in the transcriptome). *Ae. aegypti* OR name ID columns list the OR name in *Ae. aegypti* and the VectorBase code (AAELxxxxxx) of *Ae. aegypti* orthologs, respectively, with the percent (%) of identity reported in the last column

**Fig. 6:**
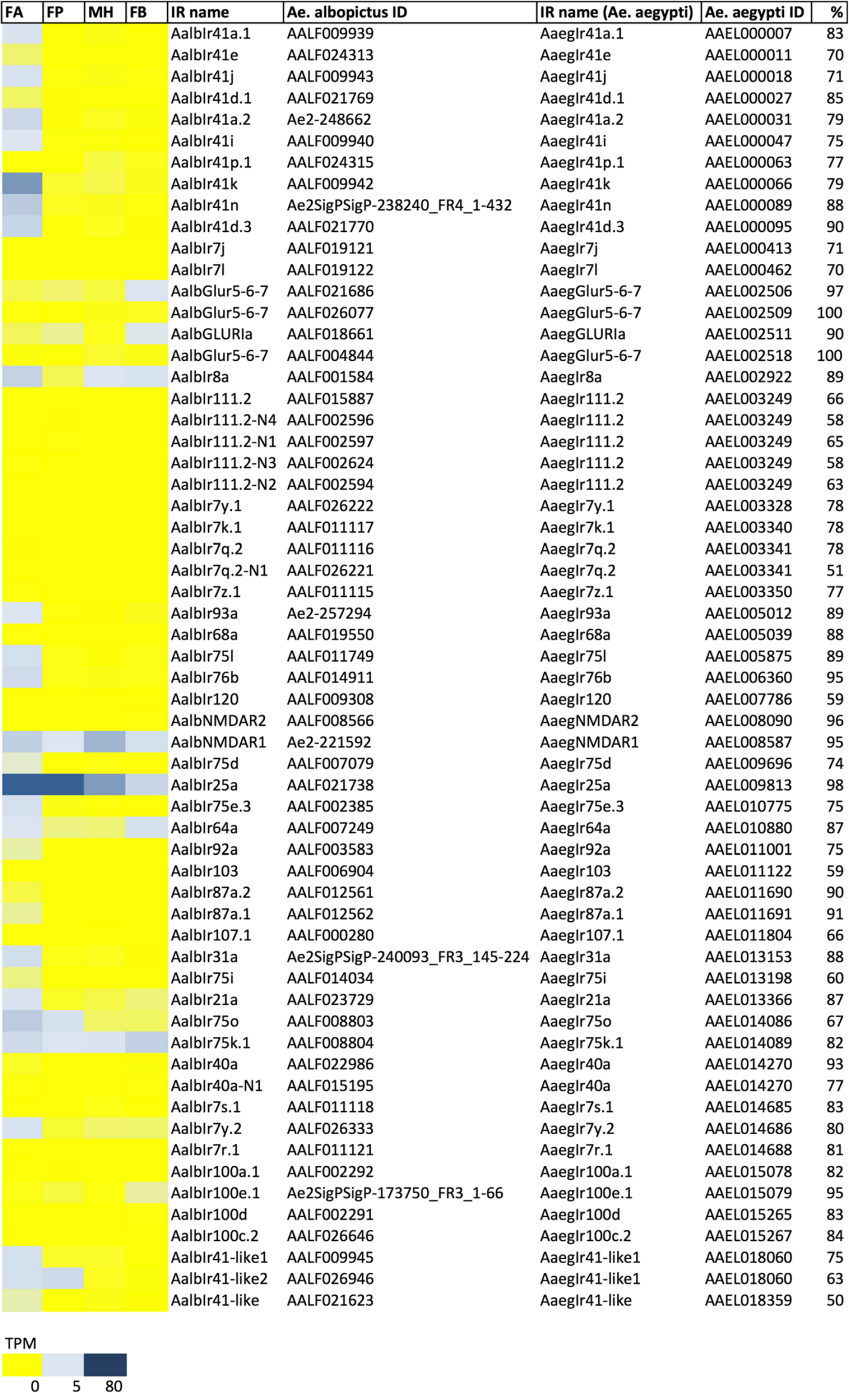
Ionotropic receptors (IR) in *Ae. albopictus* transcriptome. Left panel, abundance profile map: intensity scale (color gradient from blue to yellow indicates levels from high TPM to low) as indicated at the bottom. FA, female antennae; FP, female palps; MH male heads and FB, female body. IR name and *Ae. albopictus* ID columns indicate respectively the name assigned to IR in *Ae. albopictus* and the ID (VectorBase ID AALFxxxxxx when available or contig ID in the transcriptome). *Ae. aegypti* IR name and ID columns list the IR name in *Ae. aegypti* and the VectorBase code (AAELxxxxxx) of *Ae. aegypti* orthologs, respectively, with the percent (%) of identity reported in the last column

**Fig. 7:**
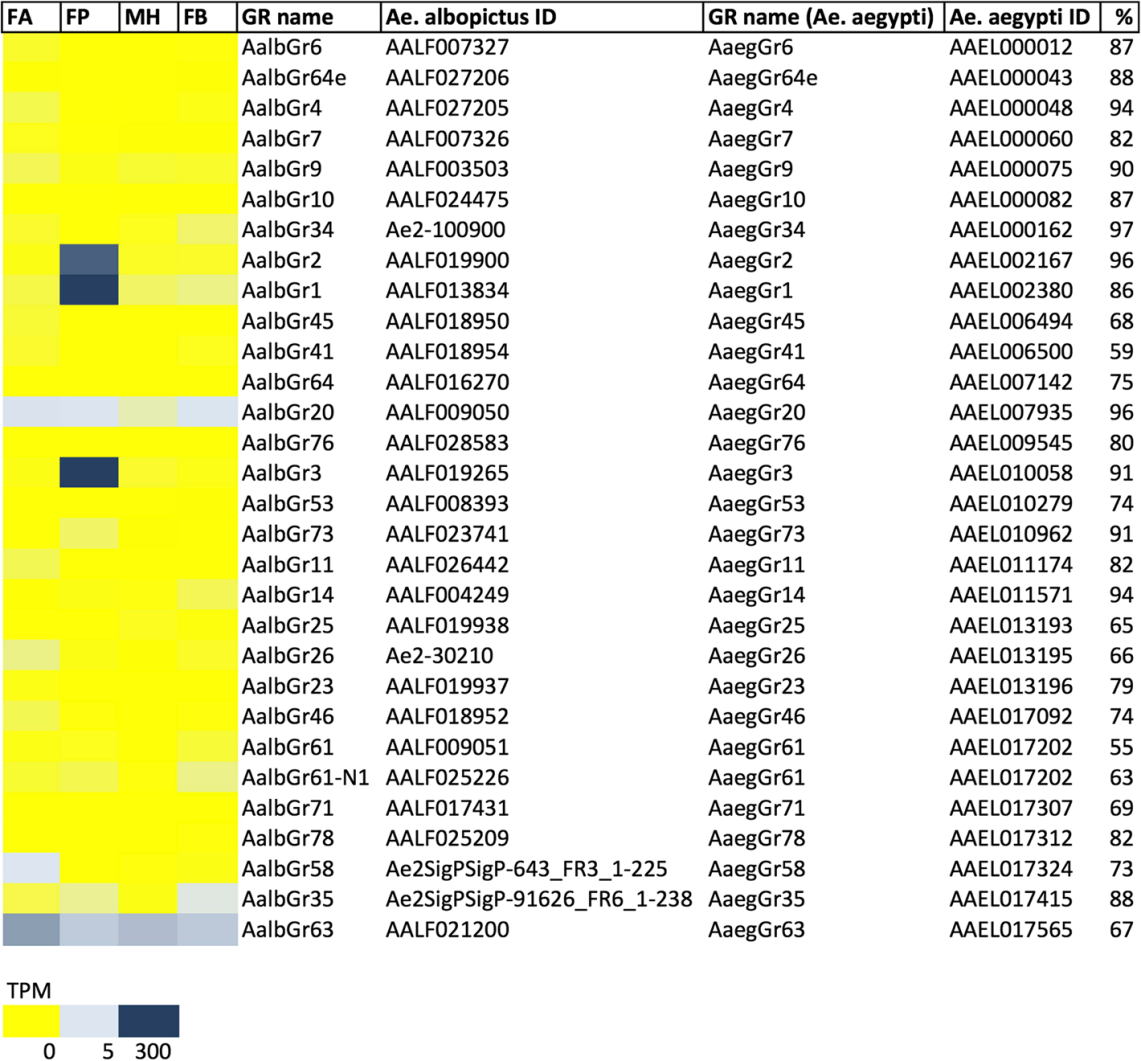
Gustatory receptors (GR) in *Ae. albopictus* transcriptome**.** Left panel, abundance profile map: intensity scale (color gradient from blue to yellow indicates levels from high TPM to low) as indicated at the bottom. FA, female antennae; FP, female palps; MH male heads and FB, female body. GR name and *Ae. albopictus* ID columns indicate respectively the name assigned to GR in *Ae. albopictus* and the ID (VectorBase ID AALFxxxxxx when available or contig ID in the transcriptome). *Ae. aegypti* GR name and ID columns list the GR name in *Ae. aegypti* and the VectorBase code (AAELxxxxxx) of *Ae. aegypti* orthologs, respectively, with the percent (%) of identity reported in the last column

Chemosensory pathways in vector mosquitoes have been extensively characterized through genomics and transcriptomics studies in the last 10 years providing the scientific community with comprehensive lists of olfactory genes from *An. gambiae*, *Ae. aegypti* and *C. quinquefasciatus* [38, 69–71, 74–77]. We have searched the transcriptome profiles for genes with products that are involved in sensory functions by blasting the contigs against several databases (as, for instance, Invertebrate and Diptera protein databases), as detailed in the Methods section. We have also used published datasets of *Ae. aegypti* chemosensory genes as a query (ORs: [69–71]; GRs [22, 69, 71]; IRs: [27, 69, 71]; OBPs: [38, 69, 71]) and a reciprocal BLASTP algorithm to identify true orthologs in our *Ae. albopictus* transcriptome. Finally, the lists were further extended by manually guided reciprocal BLAST that allowed including contigs showing high similarity over shorter regions. Lists of 77 OBPs, 82 ORs, 60 IRs and 30 GRs were obtained (Table 6 and Figs. 4-7). As detailed below, several members of each family showed specific or enriched abundance in one or both olfactory appendages (Additional file 10: Table S1 and Figs. 3-7).

**Table 6:** Chemosensory gene families

	Transcriptome (33,846)	Antennae (5060)	Palps (5197)	Male Heads (5055)
OBP	77 (68)	30	26	15
OR	82 (59)	52	11	3
IR	60 (43)	25	6	6
GR	30 (28)	2	4	0
Tot.	249	109	47	24

Table 6**.** Chemosensory gene families. In the column Transcriptome is reported the number of genes belonging to each chemosensory family identified in the transcriptome upon manual and automated (in brackets) BLAST analysis. In the other columns (Antennae, Palps and Male Heads) are reported the number of chemosensory genes in each enriched subset (see Table 2, threshold at FDR < 0.05)

#### Odorant binding proteins

Taken together our RNA-seq analysis indicates that *Ae. albopictus* transcriptome includes at least 77 OBPs carrying amino acid signatures characteristic of several different OBP sub-families and clusters as described in [38] (Fig. 4, Additional file 11: Figure S3, Additional file 12: Figure S4, Additional file 13: Figure S5, Additional file 14: Figure S6). Accordingly, these could be classified as Classic OBPs (32), PlusC OBPs (16) and Atypical OBPs (26). In addition, 3 OBPs could be classified as MinusC, lacking cysteines C2 and C5 (Fig. 4). The evolution of the MinusC subfamily in Holometabola is quite intriguing, since members of this sub-group were found in the Drosophilidae, Bombyx/Tribolium, and Apis lineages as well as in the genomes of *Ae. aegypti* and *C. quinquefasciatus* but not in *An. gambiae* [38]. A possible link to the Atypical/Two domains subfamily is provided by the observation that the “matype2” group (name of this OBP cluster as defined in [38]) is characterized by a first OBP-like domain lacking C2 and C5 cysteines [38]. Four of the putative OBP proteins identified (AalbOBP20/59-AALF005057, AalbOBP25/24-AALF018602, AalbOBP26/23-AALF018601 and AalbOBP63/42-Ae2SigPSigP-217188_FR2_96–312) show the same percentage of identity with multiple *Ae. aegypti* OBPs. Moreover, two contigs (AALF022642 and AALF020568) showing high sequence similarity with OBP domains were unusually long and therefore have been manually annotated. They were probably chimeric genes wrongly built during the assembly step: the novel putative sequences are reported in Additional file 15: Dataset S5.

The intensity range of OBP transcript abundance is broad, spanning from 0 to 33,100 TPM. As expected, most transcripts encoding Classic OBPs displayed enhanced abundance in female antennae (heat map in Fig. 4), while PlusC family members showed a less defined profile with some OBP specifically enhanced in female palps (e.g. AalbOBP51 and AalbOBP70). Atypical (Two domains) OBPs were generally expressed at low levels in all samples analysed, with the only exception of AalbOBP107, which is the only Atypical OBP with TPM > 1. Classic and PlusC OBP transcripts were also robustly abundant in male heads. Although our dataset does not include female heads, which would give indication regarding non-chemosensory neuronal tissues, it is likely that at least some of these proteins (especially belonging to the Classic sub-family) may be involved in physiological processes and perhaps behaviours shared between the two sexes (Additional file 10: Table S1). Among contigs enhanced in the antennae, AALF023075 encodes for the putative homolog of *An. gambiae* OBP1 (AGAP003309, 78% identity) that was shown to mediate the binding with the attractant indole [41]. Recent functional studies based on the analysis of binding affinities for physiologically relevant compounds showed a certain degree of selectivity for a few mosquito OBPs, able to preferentially carry certain ligands than others [39, 41, 78]. Taken together, the transcriptome profiles presented here are consistent with previously published transcriptomic datasets of *Ae. aegypti* palps [69] and antennae [71, 79]. A total of 28 OBPs were previously described in *Ae. albopictus* [80–82] and are summarized in Additional file 16: Table S2. A comprehensive list of *Ae. albopictus* putative OBP (86) was predicted from the genome sequence and validated by RNA-seq analysis of developmental stages [62]: a detailed comparison with OBP from our transcriptome is reported in Additional file 17: Table S3.

#### Odorant receptors

Our analysis identified 82 contigs encoding putative ORs in the combined transcriptome profile of *Ae. albopictus* chemosensory tissues. Within a threshold of TPM > 3 in at least one sample, 49 OR transcripts may be classified into three broad categories. Most ORs (39) are enriched in the female antennae (using as sorting criteria, those with TPM > 3 in the antennae, TPM < 3 in the other tissues and at least 10-fold TPM increase in the antennae compared to palps and/or male heads). Among these, the homologue (79% identity) of AaegOR4 was identified in *Ae. albopictus* and showed a comparable intensity and specificity of expression in the female antennae [71, 79]. Recent studies in *Ae. aegypti* found AaegOR4 enriched in the antennae of *Ae. aegypti* mosquitoes with an anthropophilic host preference therefore suggesting this OR could play an important role in establishing that important behavior [19]. A second group comprising 6 ORs displayed a more ubiquitous abundance profile and, not surprisingly, this includes the transcript for the obligate ORco co-receptor that is expressed in all ORNs where it is required for establishing functional OR-complexes [6]. Finally, three ORs (AalbOR8, AalbOR49 and AalbOR91) are highly enriched if not specific to *Ae. albopictus* maxillary palps Fig. 3 and Fig. 5, which aligns to similar patterns already described for their orthologs in *An. gambiae* [24, 83] and *Ae. aegypti* [69]. Indeed, homologues of AalbOR8 show enhanced expression profiles in *Ae. aegypti* and *An. gambiae* maxillary palps, as the homologue of AalbOR49 in *Ae. aegypti* is similarly enriched in the palps [24, 69]. In *An. gambiae* AgOR8 is expressed in maxillary palp OSNs that are highly sensitive to 1-octen-3-ol [24], a chemical component contained in human breath and sweat, one of the factors responsible for the attraction to human host. Interestingly, *Ae. albopictus* mosquitoes are similarly attracted by 1-octen-3-ol [84].

Beyond these, 25 contigs encode putative ORs that display low sequence identity (< 70%) with cognate *Ae. aegypti* orthologs; some of these may be novel *Ae. albopictus* receptors resulting from gene duplication and subsequent evolutionary divergence. As in Anophelines [85], the rapid gain (and loss) of ORs that belong to chromosomal gene clusters is a hallmark of species-specific evolution. This can be inferred especially when multiple putative *Ae. albopictus* orthologs (with more than ~70% identity) are found by BLASTP searches using the same *Ae. aegypti* OR as query, and quite a few examples (9 AAELxxxxxx queries match with more than one AALFxxxxxx/contig) can be found in Fig. 5. Among these, *Ae. aegypti* OR61 (AAEL017277) identifies 4 putative homologs in the *Ae. albopictus* transcriptome (Additional file 18: Figure S7). However, only AALF018937/AalbOR61 was relatively abundant (5 < TPM < 80) in all samples analysed and may be considered the true ortholog of AaegOR61 also in view of the higher identity (79%). The remaining three (AALF018936/AalbOR61-N1, AALF003919/AalbOR61-N2 and AALF003920/AalbOR61-N3) are most likely the result of gene duplication, show lower transcript abundance (5 < TPM) in all samples and their possible role needs further investigation. A similar situation is found for AaegOR70 (AAEL001224) with two hits in the *Ae. albopictus* transcriptome: (i) Ae2SigPSigP-43314_FR6_155–284/AalbOR70, which is rather abundant in all samples and shows higher identity (79%) to AaegOR70 and (ii) AALF019061/AalbOR70-N1, which is enriched in female antennae and shares 64% identity with AaegOR70.

Further studies will be necessary to validate the hypothesis that gene duplication events took place in the *Ae. albopictus* OR gene families. Antennal transcriptomic analysis of two sibling Anophelinae species with different feeding behaviour showed that while their OR repertoires were highly conserved, the rates of evolution of each chemosensory gene family were more rapid than the genomic background and, importantly, there were species-specific shifts in OR transcriptome profiles [86]. Moreover, while there is an overall conservation in OR gene numbers among 16 anophelinae species, probably because of their role in several critical behaviours, numerous examples of gene gain and loss in specific anophelinae lineages were noted, reflecting the importance of ORs in the functional divergence and acquisition of novel features in the evolution of different behavioural traits (feeding behaviour, mating, oviposition site choice, etc.) [85]. A few *Ae. albopictus* ORs have been previously characterized: the female antennae-specific AalOR2 [87] renamed as AalbOR2 in this study, the *Ae. albopictus* ORco co-receptor and a subset of tuning AalbORs already published [88]. Moreover, a list of predicted OR was deduced from the genome sequence and validated by RNA-seq analysis of developmental stages [62]: the comparison with OR described in our transcriptome is shown in Additional file 17: Table S3.

#### Ionotropic receptors

Sixty contigs encoding putative *Ae. albopictus* IRs (AalbIRs) were identified, most of which enriched in female antennae (Fig. 3 and Fig. 6). Setting the threshold at TPM > 3 in at least one tissue, 29 putative AalbIRs could be identified. Among these 15 AalbIRs are either specific to the female antennae or display at least a 10-fold increase in raw TPM when compared to male heads and female palps. A second group of at least 8 AalbIRs showed similar abundance in most if not all samples analysed and, not surprisingly, they include the *Ae. albopictus* orthologs of IR25a and IR8a which were shown to act as IR co-receptors in both Drosophila [28, 89–91] and more recently *An. gambiae* (Pitts et al. 2017). It is worth noticing that IR25a and IR8a transcripts are highly abundant in antennae and/or maxillary palps of *Ae. albopictus, Ae. aegypti* and *An gambiae* mosquitoes [69, 71, 83]; instead in *Culex quinquefasciatus* while IR8a was found the most abundant IR expressed in the antennae [92], IR25a was absent [93]. Finally, AalbIR76b, which is the homologue of the third IR co-receptor IR76b, recently shown to be required to detect the smell and taste of polyamines in the fruit fly [31], is only moderately abundant in the *Ae. albopictus* female antennae. In contrast, IR76b is expressed at high levels in both *An. gambiae* antennae and maxillary palps [74, 94], in the antennae, palps and labellum of *Ae. aegypti* [69, 71, 79], as well as is found enriched in the antennae of *C. quinquefasciatus* [93].

Beyond these co-receptors, thirteen members of the IR41 sub-family of receptors are found in the transcriptome, with most of them (11/13) showing an enriched presence in the antennae sample. IR41a and IR41c have been recently shown to bind the heterocyclic amine pyrrolidine in *An. gambiae* [29]. Moreover, studies in the fruit fly demonstrated that IR41a, co-expressed with IR76b, mediates odour attraction, detecting polyamines present in fruits and in specific oviposition sites [31]. Interestingly, polyamines are also responsible for the attraction of *Ae. aegypti* mosquitoes to egg-laying sites [31].Two putative orthologs of AgIR41a are found in *Ae. albopictus*, AALF009939 (AalbIR41a.1) and Ae2–248,662 (AalbIR41a.2) showing 56% and 44% identity, respectively. Another possible gene duplication event may be described for IR41p.1, which is encoded by two genes in *Ae. aegypti*: AAEL000063 and AAEL018060. In *Ae. albopictus*, AalbIR41p.1 (AALF024315) shows a good level (77%) of identity with *Ae. aegypti* AAEL000063 (Fig. 6 and Additional file 19: Figure S8) and presents a weak expression profile (only in male heads TPM > 1). AAEL018060 has two putative orthologs in *Ae. albopictus*, showing different expression profiles: AALF009945 (AalbIR41-like1) that is specifically expressed only in female antennae (TPM = 8,82 and TPM < 1 in the other tissues) and AALF026946 (AalbIR41-like2) that is specific of both female antennae and palps. Other putative gene duplication events were found while using the *Ae. aegypti* AaegIR111.2 (AAEL003249) as query to search our transcriptome. Indeed, five *Ae. albopictus* homologs (58 to 66% identity) expressed at low abundance in olfactory organs (TPM < 1) and four of which arranged in cluster were identified in our olfactory transcriptome (Fig. 6, Additional file 20: Figure S9B). Moreover, as shown in Additional file 20: Figure S9A (from VectorBase), ten putative *Ae. albopictus* homologues of AaegIR111.2 and of the 75% identical AAEL018094 are predicted by the Comparative Gene Tree tool in VectorBase (VBGT00730000019944). Finally, another example of genetic cluster rearrangement was analysed in detail for IR7. In *Ae. albopictus*, at least 10 contigs belonging to the IR7 family were found. They show a generally weak expression profile; seven of these are clustered in a ~50 kb region and careful analysis indicated that two additional family members with no evidence of expression may be part of the same cluster (Additional file 21: Figure S10). Comparison to the *Ae. aegypti* genomic scaffold containing the IR7 cluster reveals a certain degree of gene shuffling among family members previously annotated in *Ae. aegypti*, with six orthologs identified in a 100 kb region (Additional file 21: Figure S10 and Additional file 15: Dataset S5).

#### Gustatory receptors

We identified 30 distinct contigs encoding putative GR in the transcriptome of *Ae. albopictus* two of which (AalbGR20 and AalbGR63) are modestly abundant across all samples. Furthermore, as expected, three putative GRs (AalbGR1, AalbGR2 and AalbGR3) which are orthologous to a triad of GRs expressed in the maxillary palp of *An. gambiae* [24] and *Ae. aegypti* females [69] were similarly differentially abundant in the palps of *Ae. albopictus* female (Fig. 3 and Fig. 7). Functional studies revealed a central role of both AaegGR1 and AaegGR3 in the perception of CO_2_ [25, 26] which plays a crucial role in mosquito perception of both heat and the attractant lactic acid in *Ae. aegypti* thus supporting the hypothesis that multimodal integration of CO_2_, heat, and odours mediates host-seeking and blood-feeding behaviour [26]. The high degree of identity of AalbGR1 and AalbGR3 with their *Ae. aegypti* homologues (86% and 91%, respectively) along with data from field studies revealing that *Ae. albopictus* mosquitoes respond to attractants l-octen-3-ol (octenol) and L-lactic acid [84], lead us to hypothesize that AalbGR1 and AalbGR3 are likely to be components of *Ae. albopictus* maxillary palp CO_2_ receptor complex. In addition, only a single putative GR (AalbGR58) was found to be specifically expressed and relatively abundant (TPM > 3) in *Ae. albopictus* female antennae. Three other GRs (AalbGR20, AalbGR35 and AalbGR63) showed an ubiquitous presence in different samples of the mosquito. According to recent transcriptomic catalogues, homologues of these three receptors were not transcriptionally active in antennae and palps of *Ae. aegypti* mosquitoes [69, 71]. Sequence homology among these seven abundant GRs spans between 5.9 and 37%, as reported in Additional file 22: Table S4.

### Other contigs overexpressed in sensory organs

**Fig. 8:**
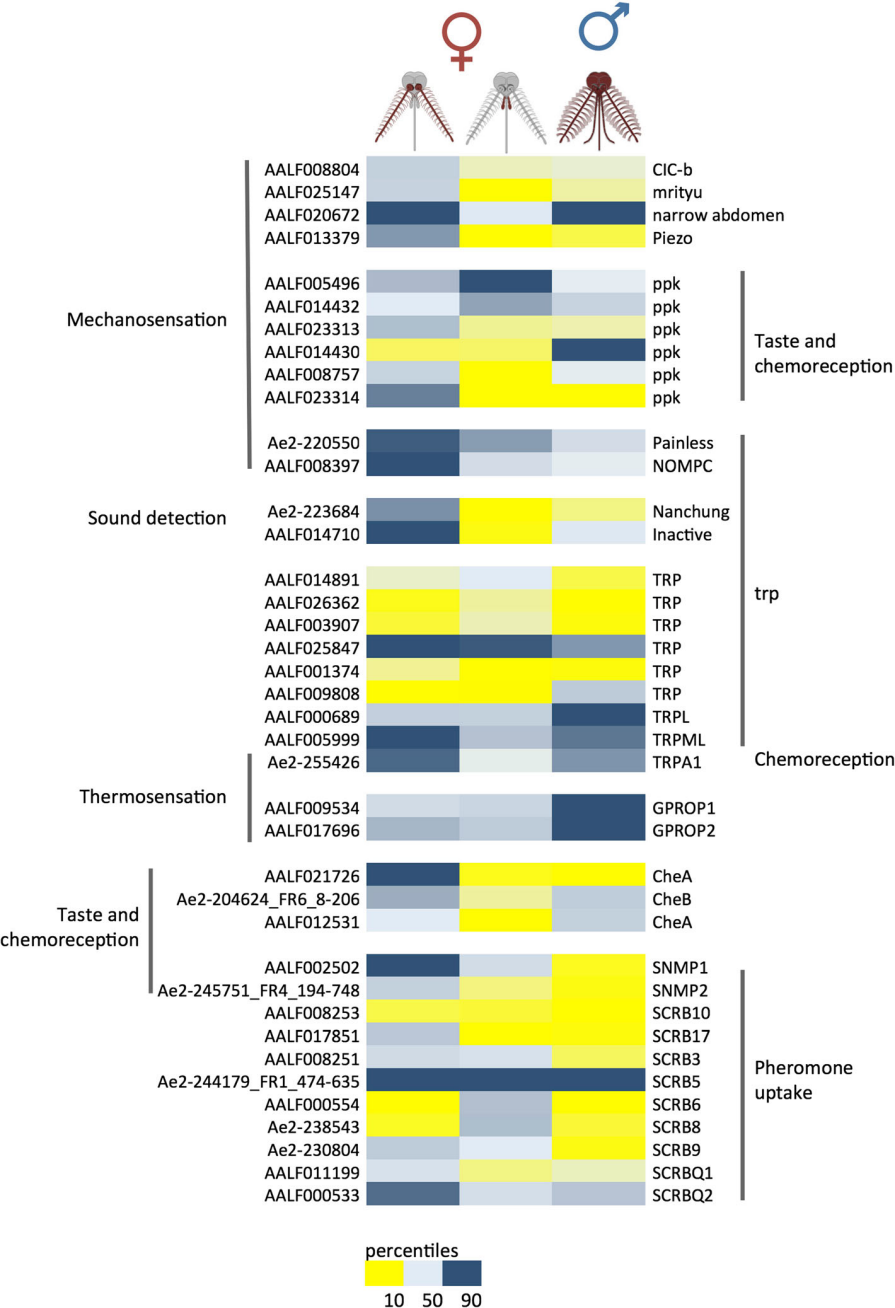
Abundance profiles of sensory genes in *Ae. albopictus*. Abundance profile map: intensity scale (color gradient from blue to yellow indicates levels from high TPM to low) as indicated at the bottom. Gene name and contig ID (VectorBase codes when available) are also reported

In addition to the OBP, OR, IR and GR gene families, which are often labeled as the “main chemosensory components”, several contigs encoding putative proteins involved in olfaction are found among those differentially expressed in antennae and palps (FDR < 0.001, Fig. 8). Contigs encoding for putative odorant and pheromone-degrading enzymes (ODEs and PDEs), that play essential roles in clearing sensillar lymph, were identified. Among these, members of four relevant subfamilies were found: (i) cytochrome P450 (CYPs), with 8 and 2 contigs in the antennae-and palps-specific clusters, respectively [95, 96]; (ii) esterases (ESTs) with 1 contig in the antennae- and 3 contigs in the palps-specific clusters [97, 98]; (iii) hydroxysteroid dehydrogenases (HSD), also involved in the degradation of hormones [99], with 3 and 4 contigs in the antennae- and palps-specific clusters, respectively; (iv) glutathione S-transferase (GST), which has also been implicated in odorant clearing and degradation pathways [100, 101] and, according to TPM values, it is one of the most abundant contigs in the antennae-enriched list. It is worth mentioning that insect GSTs are generally involved in the protection against oxidative stress, detoxification of both endogenous and xenobiotic compounds and are also entailed in intracellular transport, biosynthesis and metabolism of hormones. Moreover, a crucial role of insect GSTs in insecticide resistance was also proved [102, 103].

Beyond ODEs/PDEs other contigs were found significantly enriched in antennae and palps. One of the most abundant female antennal contig is AALF015401, which encodes a conserved secreted protein whose putative ortholog in *An. gambiae* (AGAP007976) is also antennae-specific and encodes a protein with as yet no function. Another antennae-enriched contig (AALF007737) encodes for a putative peptide showing similarity with antennal carrier protein AP-2 [104]. Finally, several additional antennae-enriched contigs coded for putative cuticular proteins [105], core cytoskeletal components (dynein heavy chains), juvenile hormone-inducible protein and hemolymph juvenile hormone binding protein. Some of the ODEs (esterases, cytochrome P450) as well as juvenile hormone-inducible proteins were among the most abundant contigs found in the palps. Moreover, AALF013835 that encodes for a potassium/sodium hyperpolarization-activated cyclic nucleotide-gated channel (HCN) showed high and specific expression in *Ae. albopictus* palps where, in light of the odorant-gated ion channel based activity of insect chemosensory neurons, it likely plays a modulatory role in maintaining neuronal membrane potential (and therefore action potential frequency) [106]. Finally, contigs encoding for proteins possibly involved in immune defense mechanisms were found in both antennae- and palps-specific clusters. In particular, palps-specific contigs encoding for leucine rich repeats protein (LRR, AALF017691), prophenoloxidase (PPO, AALF012718), cecropin C (CEC, Ae2SigPSigP-213035_FR1_18–90), and for a member of the nimrod family (AALF007372) were identified.

### Genes involved in other sensory modalities

In addition to the sensory genes described so far, several other protein families play crucial roles in insect sensing of the external environment. These proteins are involved in different physiological pathways such as mechanosensation, thermosensation, perception of taste, light, humidity and gravity, sound detection and other forms of stimuli. A selection of these sensory genes and their abundance profiles is shown in Fig. 8, that highlights also their wide functional overlap, with members of a gene family often involved in more than one perception pathway (see ppk family members taking part to both mechano- and taste/chemo-perception). We will describe some of the most relevant protein families involved in i) taste- and chemo-perception, ii) mechanosensation and iii) thermosensation.

#### Chemo- and taste-perception

Members of the CheA/CheB gene family are small and secreted proteins mainly involved in perception of chemical stimuli and most of them interact with ppk channels. Using a list of 10 *Ae. aegypti* CheA and 3 CheB sequences [69, 79] we have identified 17 *Ae. albopictus* contigs. Of these, only 3 CheA/B transcripts (AALF012531, AALF021726 and Ae2-204624_FR6_8–206) display TPM > 1 in antennae and/or palps. In particular, AALF021726 (orthologs of AAEL012061) is by far the most abundant (TPM > 10) and specific CheA of female antennae, differently from its *Ae. aegypti* ortholog (TPM < 1 in the antennae, [71]). Ae2-204624_FR6_8–206 (ortholog of AAEL009978, very scarce in *Ae. aegypti* antennae, TPM < 1) is abundant in female antennae (TPM = 3.6) and to a lesser extent in the palps (TPM = 1.0) (Fig. 8 and Additional file 23: Table S5). Both CheA/CheB are highly expressed in the tarsi of *Ae. aegypti* mosquitoes [23] and at a lesser extent in maxillary palps [69]. Moreover, orthologs of AALF021726 and Ae2-204624_FR6_8–206 in *Ae. aegypti* mosquitoes are the most abundant members of CheA and CheB expressed in the labella [23]. The third member of *Ae. albopictus* CheA family (AALF012531), putative ortholog of AAEL004928, was found expressed in female antennae and male heads.

Members of the *pickpocket* gene family (degenerin/epithelial Na^+^ channel, DEG/ENaC) and members of the *trp* family (transient receptor potential channels) are associated with taste perception, photo-, thermo-, and mechanoreception [46]. In mosquitoes, maxillary palps and proboscis are head appendages that along with the tarsi and labella play an essential role in gustatory pathways [23, 69, 79, 107]. We have identified 18 ppk receptors in the *Ae. albopictus* transcriptome (Additional file 23: Table S5). Of these, 5 contigs have a TPM value >1 (Additional file 23: Table S5) in at least one sensory appendage library, with two abundance patterns: i) contigs enhanced in antennae vs palps (AALF023313, AALF023314 and AALF008757); ii) contigs that are more abundant in female maxillary palps than antennae (AALF005496 and AALF014432).

SNMPs belong to the scavenger receptor type B gene family (SCRB/CD36) [32, 33], are involved in a more wide range of functions. Using 13 *Ae. aegypti* putative SCRB genes as query we identified 12 putative orthologs (identity >73%) in *Ae. albopictus*. SNMP1 was strongly enhanced in female antennae, while SNMP2 showed a more ubiquitous expression profile (Additional file 23: Table S5 and Fig. 8) as also reported in *Ae. aegypti* [79]. Among the 11 other SCRBs found in *Ae. aegypti* sensory organs [69], we identified 10 orthologs in *Ae. Albopictus*. Overall, they are abundantly expressed in head chemosensory appendages, with SCRB5 and SCRB6 highly enriched in maxillary palps (TPM = 923 and TPM = 40, respectively), similarly to *Ae. aegypti* [71]. The exact role of these proteins in head organs is unknown, however, they are well conserved among most insect species and may also have non-chemosensory roles [34].

#### Thermosensation

Several members of the TRP gene family, composed by 13 genes belonging to 7 subfamilies, are involved in temperature perception and other aspects of insect behaviour, including avoidance of noxious heat/odorants/tastants [46]. Noxious heat perception is most likely carried out by antennae as reported in *Ae. aegypti* [108] and *An. gambiae* [48]. TRPA1 is a heat-activated channel expressed in thermosensitive sensilla of *An. gambiae* female antennae [48]. We have identified TRPA1 ortholog in *Ae. albopictus* and several contigs with high level of similarity to other *Ae. aegypti* TRP family members. *Painless* (another TRPA family member) also associated with avoidance of dry climates and mechanosensory stimuli was shown to be expressed in *Ae. albopictus* antennae and palps. Finally, we have identified the putative homolog of *Ae. aegypti* TRPA family gene *pyrexia* in *Ae. albopictus* transcriptome albeit with low level of abundance in sensory organs.

Members of rhodopsin family of GPCRs (G protein–coupled receptors) are known to play a central role in photoreception (vision) [6], however, they may be also involved in the perception of temperature, since an overlap between genes involved in light detection and in temperature discrimination is known in insects [109]. Most of the *Ae. albopictus* GPROP family members are highly abundant in male heads, as expected with their main role in vision. GPROP1 and GPROP2 are also highly abundant in female antennae and palps (Additional file 23: Table S5), whereas only basal levels were observed for other members of the family (GPROP3, GPROP5, GPROP8 and GPROP9). The arrestin family is a small group of genes involved in the termination and desensitization of GPCR-based transduction as well as a range of downstream cellular signalling pathways [110]. We identified *Ae. albopictus* arrestin1 and arrestin2 genes, that, as expected, were overexpressed in male heads, which likely reflects their role in the ommatidia of insects where they have been shown to quench the photo response by directly interacting with photo-activated rhodopsin GPCRs [111, 112]. Arrestin1 and arrestin2 have also been characterized in olfactory tissues of both *Drosophila* and *An. gambiae* [113], and are not surprisingly detected also in the maxillary palps of *Ae. aegypti* mosquitoes [69], providing a rationale for our detection of the *Ae. albopictus* ortholog for arrestin1 expression in female palps and antennae.

#### Mechanosensation

Transduction of mechanical stimuli plays an essential role in many aspects of mosquito life cycle that include hearing, touch and pain sensitivity [6]. *Ae. albopictus* piezo, an evolutionarily conserved transmembrane protein involved in mechanosensory transduction in Drosophila [51] is relatively abundant in antennae (Fig. 8 and Additional file 23: Table S5). In *Ae. albopictus*, transcripts encoding for both piezo and the TRP channel painless are indeed abundant not only in antennae but also in female bodies, suggesting that they are also part of sensory neurons located in mosquito antennae. Other genes that have been implicated in mild touch detection appear to be specific both of female antennae, such as *nompC*, and of male sensory appendages of the head, such as *Nmdar1*. In our study, *Narrow abdomen* appears enhanced presumably in the antennae of both sexes, while *Cicb* is ubiquitous. Sound detection in *D. melanogaster* requires the TRPV channels encoded by *Nanchung* (*Nan*), *inactive* (*Iav*) and *nompC* [114] that are abundantly expressed in many head appendages [46]. Similarly to abundance profiles in *Ae. aegypti* [69, 71], we observed robust abundance of these genes in *Ae. albopictus* female antennae but not in maxillary palps, a pattern only partially overlapping with data from *An. gambiae*, where *Nanchung* and *inactive* are believed to play a role in the capacity of male antennae to detect female wing beats during mating swarms [83, 115].

### qPCR validation

**Fig. 9:**
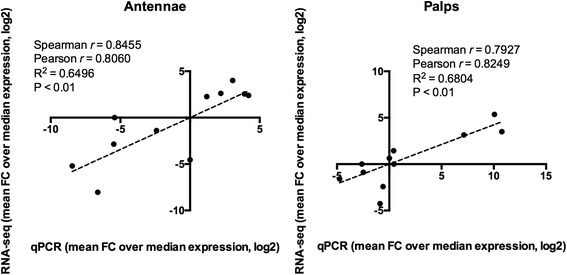
qPCR validation. Correlation between transcriptional abundance of 11 genes in both antennae (**a**) and maxillary palps (**b**) revealed by qPCR and RNA-seq. Level of abundance is defined as the ratio between each sample value over the group median (fold change, FC) in both qPCR and RNA-seq approaches. For both techniques, statistical evaluation throughout Spearman and Pearson tests was performed and results are reported in the figure insets

To validate the transcriptome profiles obtained by RNA-seq analysis, we have selected 11 genes belonging to the four main chemosensory families for analysis by quantitative real time PCR. The study was performed using independent, duplicate RNA samples extracted from female antennae and maxillary palps. Targets were chosen so as to include those with high, medium or low abundance levels in the different tissues based on our collective RNA-seq analysis. Four OBPs (AalbOBP36, AalbOBP47 and AalbOBP83, all of them highly enriched in the antennae, and AalbOBP27, expressed at the same high level – TPM > 500 – in both tissues), two ORs (AalbOR8 and AalbOR84, showing a slight enhancement of expression in palps and antennae, respectively), the ubiquitously abundant ORCO, two GRs (the palps-specific AalbGR3 and AalbGR58, that shows a weak – TPM < 10 – but specific expression in the antennae) and finally, two ubiquitous IRs, the abundant AalbIR25a and the weakly expressed AalbNMDAR1. Correlation studies indicate statistically significant (*P* < 0.01) linear relationships between datasets obtained from the two methods for both antennae and palps samples (Fig. 9). Several parameters might influence both methods, determining discrepancies and variability among abundance profiles. Even though the correlation between the two techniques is strong, a few differences can be highlighted and could provide valuable new information. In particular, it is worth mentioning AalbGR58 expression in antennae, detected at low level by RNA-seq (TPM < 10) while revealed abundant and specific by qPCR (Additional file 3: Figure S11).

## Conclusions

We present the RNA-seq generated transcriptome profiles of the main sensory appendages of the tiger mosquito *Ae. albopictus*. This study provides a tissue-specific survey of transcript abundance in adult *Ae. albopictus* sensory organs, providing the community with a first catalogue of annotated chemosensory genes. We have analyzed antennae and maxillary palps of female mosquitoes, male heads and whole female bodies as reference samples for differential expression studies. Around 350 putative proteins were classified in several functional families involved in mosquito olfaction and more generally in the perception of the surrounding environment. Our analysis also provided an important contribution for the improvement of genome annotation since most of the transcripts/proteins were manually annotated to describe molecular features and gene clusters were verified on released genomic scaffolds. In addition, *Ae. albopictus* chemosensory genes belonging to OBP, OR, IR and GR gene families were compared to homologous groups of genes identified in other mosquito species such as the malaria mosquito *An. gambiae*, the southern house mosquito *C. quinquefasciatus* and, in particular, the dengue and yellow fever mosquito *Ae. aegypti*. The comparison allowed identifying bona fide orthologs, pointing out differences in sequence and gene abundance as well as highlighting cases of gene gain/loss. Indeed, variation in abundance of olfactory/sensory genes between *Ae. aegypti* and *Ae. albopictus* may provide a first starting point to study and better understand physiological and behavioral differences between these two mosquito species.

## Additional files

Additional file 1: Alignment files. MEGA files of protein alignments and tree. (ZIP 93 kb)

Additional file 2: Table S6. Features of primers used in this study.(PDF 44 kb)

Additional file 3: Figure S11. Correlation of biological replicates. (PDF 951 kb)

Additional file 4: Dataset S1. Transcriptome: sequence features of contigs. (XLSX 12364 kb)

Additional file 5: Dataset S2. Transcriptome: comparisons with databases. (XLSX 15138 kb)

Additional file 6: Dataset S3. Transcriptome: differential expression analysis. (XLSX 14649 kb)

Additional file 7: Dataset S4. Transcriptome: sequences. (XLSX 16036 kb)

Additional file 8: Figure S1. Differential expression, cluster analysis and sample correlation matrix. (PDF 499 kb)

Additional file 9: Figure S2. GO terms enrichment. (pdf) (PDF 385 kb)

Additional file 10: Table S1. Chemosensory proteins in the different samples and average TPM. (PDF 41 kb)

Additional file 11: Figure S3. Alignment of Classic OBPs.(PDF 7646 kb)

Additional file 12: Figure S4. Alignment of PlusC OBPs. (PDF 4328 kb)

Additional file 13: Figure S5. Alignment of Atypical OBPs. (PDF 8211 kb)

Additional file 14: Figure S6. Phylogram of OBP family members in *Ae. albopictus* and *Ae. aegypti*. (PDF 125 kb)

Additional file 15: Dataset S5. Mapping of chemosensory genes on *Ae. albopictus* genomic scaffolds. (XLSX 79 kb)

Additional file 16: Table S2. Comparison with previously published ***A. albopictus*** OBP. (XLSX 14 kb)

Additional file 17: Table S3. Comparison with published ***A. albopictus*** OBP and OR in Chen X-G et al. (2015) [48]. (XLSX 67 kb)

Additional file 18: Figure S7. Alignment and phylogenetic tree of OR61. (PDF 1577 kb)

Additional file 19: Figure S8. Alignment and phylogenetic tree of IR41p1. (PDF 1841 kb)

Additional file 20: Figure S9. IR111.2 gene family in *Ae. albopictus*. (PDF 144 kb)

Additional file 21: Figure S10. IR7 genetic cluster. (PDF 250 kb)

Additional file 22: Table S4. Percentage of identity of gustatory receptors in *Ae. albopictus* sensory organs. (PDF 43 kb)

Additional file 23: Table S5. Sensory genes in *Ae. albopictus* transcriptome. (XLSX 52 kb)

## Abbreviations

CDS: Coding sequences
CEC: Cecropin C
CYP: Cytochrome P450
DEG/ENaC: Degenerin/epithelial Na + channel
EST: Esterases
FA: Female antennae
FB: Female whole body
FC: Fold change
FDR: False discovery rates
FP: Female palps
GO: Gene ontology
GPCR: G protein–coupled receptor
GR: Gustatory receptors
GRN: Gustatory receptor neuron
GST: Glutathione S-transferase
HCN: Potassium/sodium hyperpolarization-activated cyclic nucleotide-gated hannel
HSD: Hydroxysteroid dehydrogenases
Iav: Inactive
iGluRs: Ionotropic glutamate receptors
IR: Ionotropic receptors
JHBP: Haemolymph juvenile hormone binding protein
LRR: Leucine rich repeats protein
MA plot: plot using an M (log ratios) and A (mean average) scale
MH: Male heads
Nan: Nanchung
Nmdar: N-methyl-D-aspartate receptor
nompC: No Mechanoreceptor potential C
OBP: Odorant binding protein
ODE/PDE: Odorant/pheromone-degrading enzyme
OR: Odorant receptor
ORF: Open reading frame
ORN: Olfactory receptor neuron
PBP: Pheromone binding protein
PCA: Principal component analysis
PFAM: Protein family
PlcB: Phospholipase C
ppk: Pickpocket
PPO: Prophenoloxidase
qPCR: Quantitative polymerase chain reaction
SCRB/CD36: Scavenger receptor type B/CD36
SNMP: Sensory Neuron membrane protein
TPM: Transcripts per kilobase per million
TRP: Transient receptor potential channel
TSA: Transcriptome shotgun assembly
