# Supplementary material for: Deciphering the olfactory repertoire of the tiger mosquito Aedes albopictus
Source: BMC Genomics. 2017 Oct 11;18:770. doi: 10.1186/s12864-017-4144-1 (PMC5637092; doi:10.1186/s12864-017-4144-1)
Supplement: Supplementary file 2 — Features of primers used in this study.(PDF 44 kb) [file 12864_2017_4144_MOESM2_ESM.pdf]

Table S5. Properties of primers used in this study.

Primer name	Sequence	Lenght	% C-G	Tm	Product size
013336_S5_F	GTGCCACCGTGCTATAAAGTCCGT	24	54.20	64.40	101
013336_S5_R	ACGCCTTGACCACATGCTGAACGT	24	54.20	64.40	
OBP83_F	ACGTGGGCCCTCAAATGTCGCA	22	59.10	64.00	112
OBP83_R	TGCTGCTCTTGTTCTCTGCCTCGG	24	56.50	64.20	
OBP27_F	TGATGGACCTTACTCACACCGCGA	24	59.09	67.24	97
OBP27_R	AACGGCTCTCTGTAGTCATCCGGT	24	58.33	66.95	
OBP36_F	GTGGGTATCGAAATGACGGCCAGT	24	54.17	65.71	86
OBP36_R	CCGTTGAACCGAGCTATGGACTCC	24	54.17	65.26	
OBP47_F	CCACAGTGGCAACCGATAGTCAGC	24	54.17	65.02	103
OBP47_R	AGCTTGGTTTCAGCTTGGCACCGG	24	58.33	65.03	
Orco_F	ACGGCCAACACCATAACGACGCT	23	58.33	65.73	104
Orco_R	TTCCAGATTCCCAGCGTGCGGT	22	58.33	68.17	
Or8_F	GACTACGATTACAGCGCGGCGA	22	56.52	67.19	120
Or8_R	TGTGCCGAACTTGCTGCGGA	20	59.09	66.75	
Or84_F	AGCTGGCCTCTGGAAAACGGT	21	59.09	65.15	127
Or84_R	CCAGGCGAACAATGCAAAAGGGT	23	60.00	65.38	
Gr3_F	TGCGGTGTGTTCTTCATGGCACT	23	57.10	61.80	89
Gr3_R	AAGCGACCTTCCAACGTGCGGA	22	57.20	62.40	
Gr58_F	CGGCCCTAGTCTACAGAGCAAGCA	24	52.17	65.29	114
Gr58_R	TCTGCATTGACAGGGCACGCA	21	59.09	67.15	
NMDAR1_F	AGACGGTAGAGCAGTACTAGGGCG	24	58.33	66.05	86
NMDAR1_R	TGCTCTCTACAGTGGCACGAACGT	24	57.14	65.22	
Ir25a_F	TCCAACGATGAACTGGCCGCGT	22	58.33	65.09	114
Ir25a_R	CGCCTGTGCCAACTGAATGCCA	22	54.17	66.12	
