# Supplementary material for: Deciphering the olfactory repertoire of the tiger mosquito Aedes albopictus
Source: BMC Genomics. 2017 Oct 11;18:770. doi: 10.1186/s12864-017-4144-1 (PMC5637092; doi:10.1186/s12864-017-4144-1)
Supplement: Supplementary file 3 — Correlation of biological replicates. (PDF 951 kb) [file 12864_2017_4144_MOESM3_ESM.pdf]

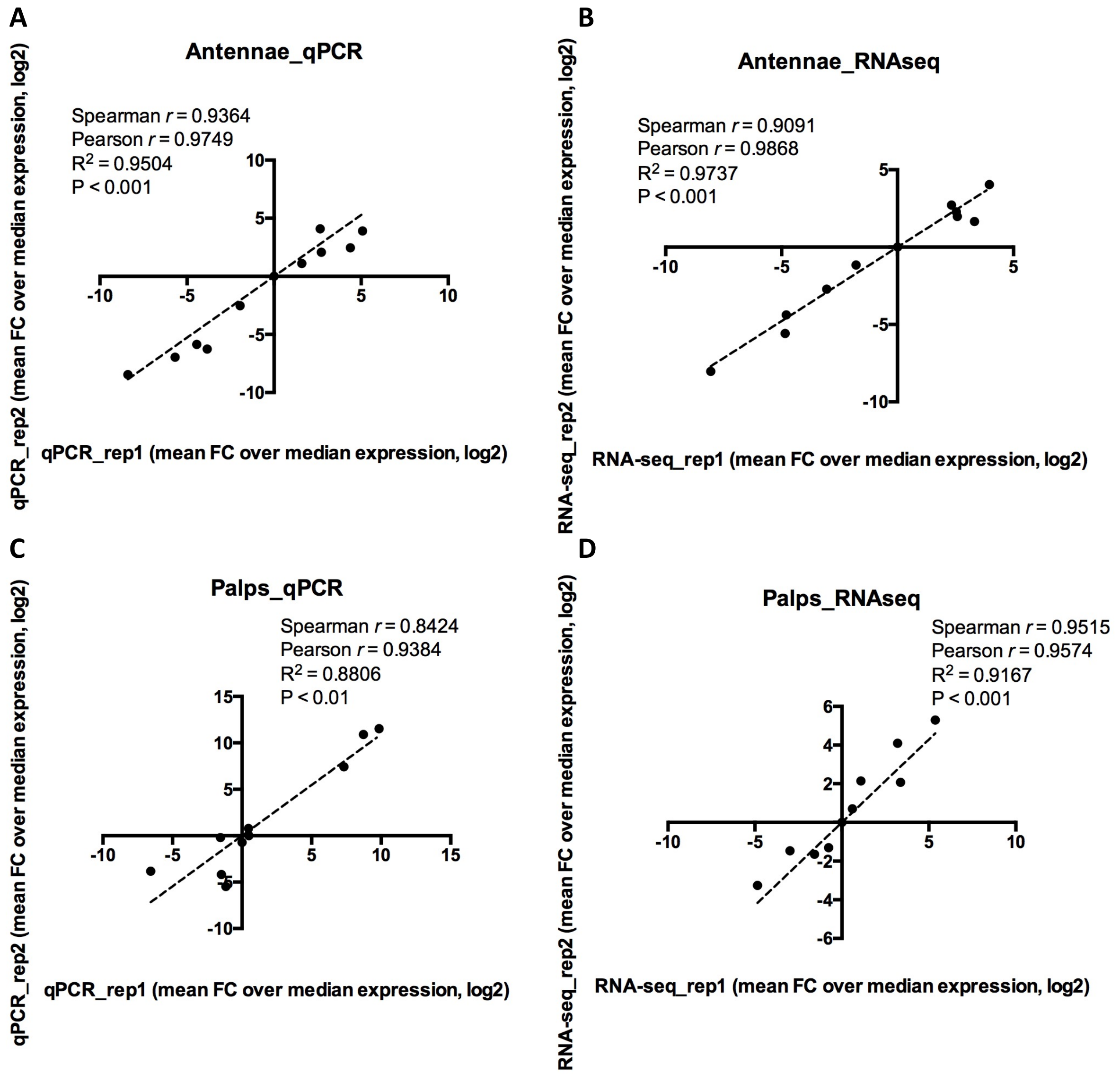

E

	qPCR				RNAseq			
	Ant_rep1	Ant_rep2	Palps_rep1	Palps_rep2	Ant_rep1	Ant_rep2	Palps_rep1	Palps_rep2
AalbOBP36	6406,691	12569,639	1,568	0,099	849,986	906,500	29,999	6,917
AalbOBP47	3948,587	4628,775	4,926	4,418	2273,526	3077,655	77,581	27,884
AalbOBP83	569,990	1801,223	0,037	0,310	870,024	737,304	17,066	5,475
AalbOBP27	1183,171	14294,343	1492,411	8496,159	734,830	1227,657	2113,392	670,105
AalbOrco	1237,824	3517,890	560,494	760,237	1462,591	589,493	526,129	72,293
AalbOr8	0,559	2,375	1,261	0,245	5,076	3,918	50,891	17,100
AalbOr84	48,629	145,658	1,202	3,869	42,390	83,985	1,765	1,791
AalbGr3	3,676	6,789	3233,049	13087,662	0,549	0,717	471,261	291,722
AalbGr58	188,549	839,491	0,000	12,649	5,243	9,000	0,000	0,000
AalbNMDAR1	8,673	14,498	3,501	2,695	17,544	28,336	6,449	6,215
AalbIr25a	13,154	10,944	4,824	7,628	145,981	186,989	109,040	75,838

10 50 90 percentiles

Figure S11. Correlation of biological replicates. Results of independent biological duplicates were correlated. Data from qPCR analysis of 11 selected genes in both antennae (A) and maxillary palps (C) are reported; data from RNA-seq analysis of the same 11 genes in both antennae (B) and maxillary palps (D) are also reported. Level of abundance is defined as the ratio between each sample value over the group median (Fold Change, FC) in both qPCR and RNA-seq approaches. For both techniques, statistical evaluation throughout Spearman and Pearson tests was performed and results are reported in the figure insets. (E) The heatmap summarizes the results obtained in each replicate in both qPCR analysis (as ng) and RNA-seq analysis (as TPM).
