# Supplementary material for: Deciphering the olfactory repertoire of the tiger mosquito Aedes albopictus
Source: BMC Genomics. 2017 Oct 11;18:770. doi: 10.1186/s12864-017-4144-1 (PMC5637092; doi:10.1186/s12864-017-4144-1)
Supplement: Supplementary file 8 — Differential expression, cluster analysis and sample correlation matrix. (PDF 499 kb) [file 12864_2017_4144_MOESM8_ESM.pdf]

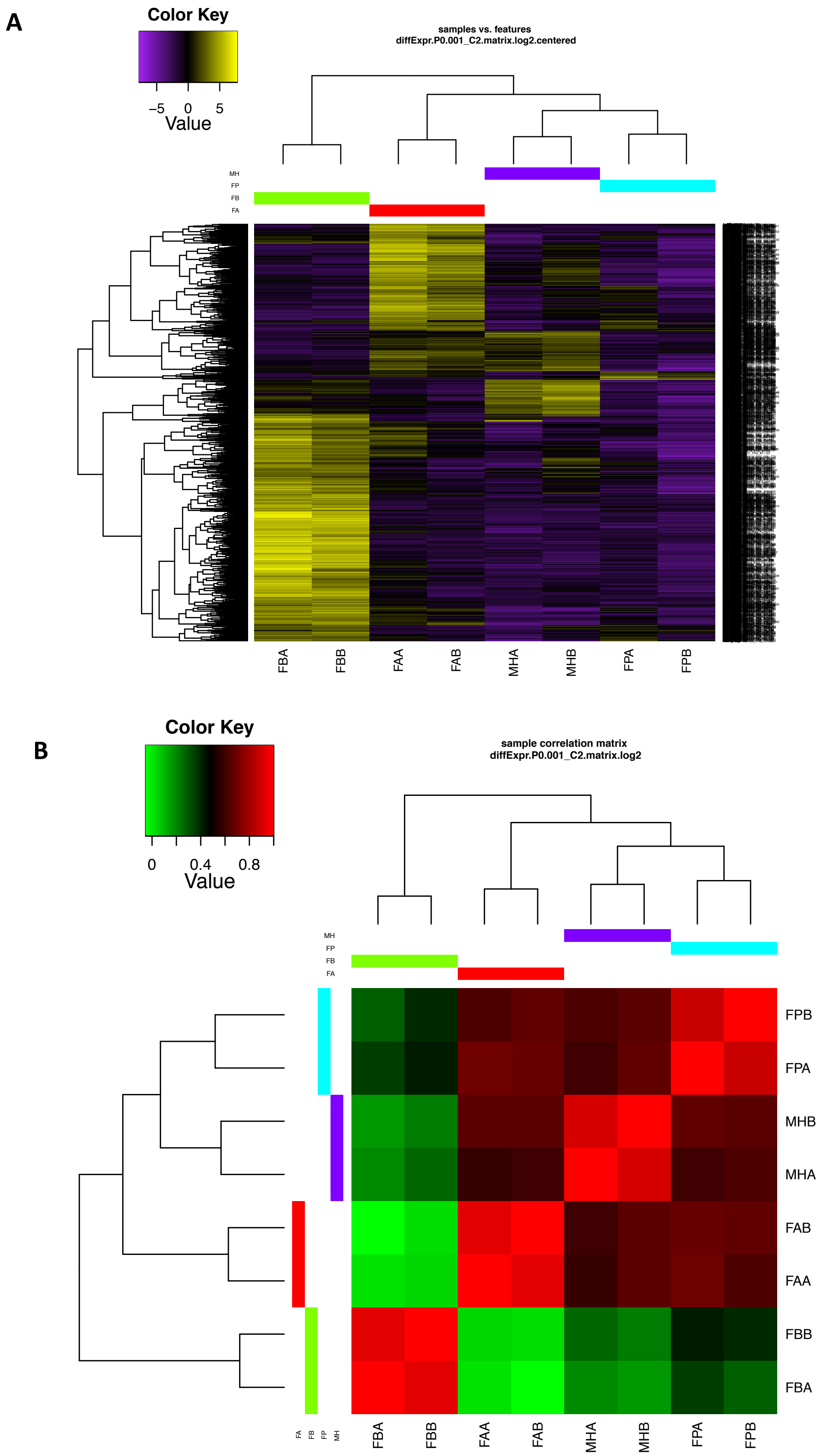

Figure S1. Differential Expression, cluster analysis and sample correlation matrix. A. Expression heatmap and cluster analysis generated according to edgeR DE analysis. Only values relative to the 3361 contigs with FDR < 0.001 and logFC >2 are shown. **B.** Correlation matrix of the different biological replicates.
