# Supplementary material for: Deciphering the olfactory repertoire of the tiger mosquito Aedes albopictus
Source: BMC Genomics. 2017 Oct 11;18:770. doi: 10.1186/s12864-017-4144-1 (PMC5637092; doi:10.1186/s12864-017-4144-1)
Supplement: Supplementary file 9 — GO terms enrichment. (pdf) (PDF 385 kb) [file 12864_2017_4144_MOESM9_ESM.pdf]

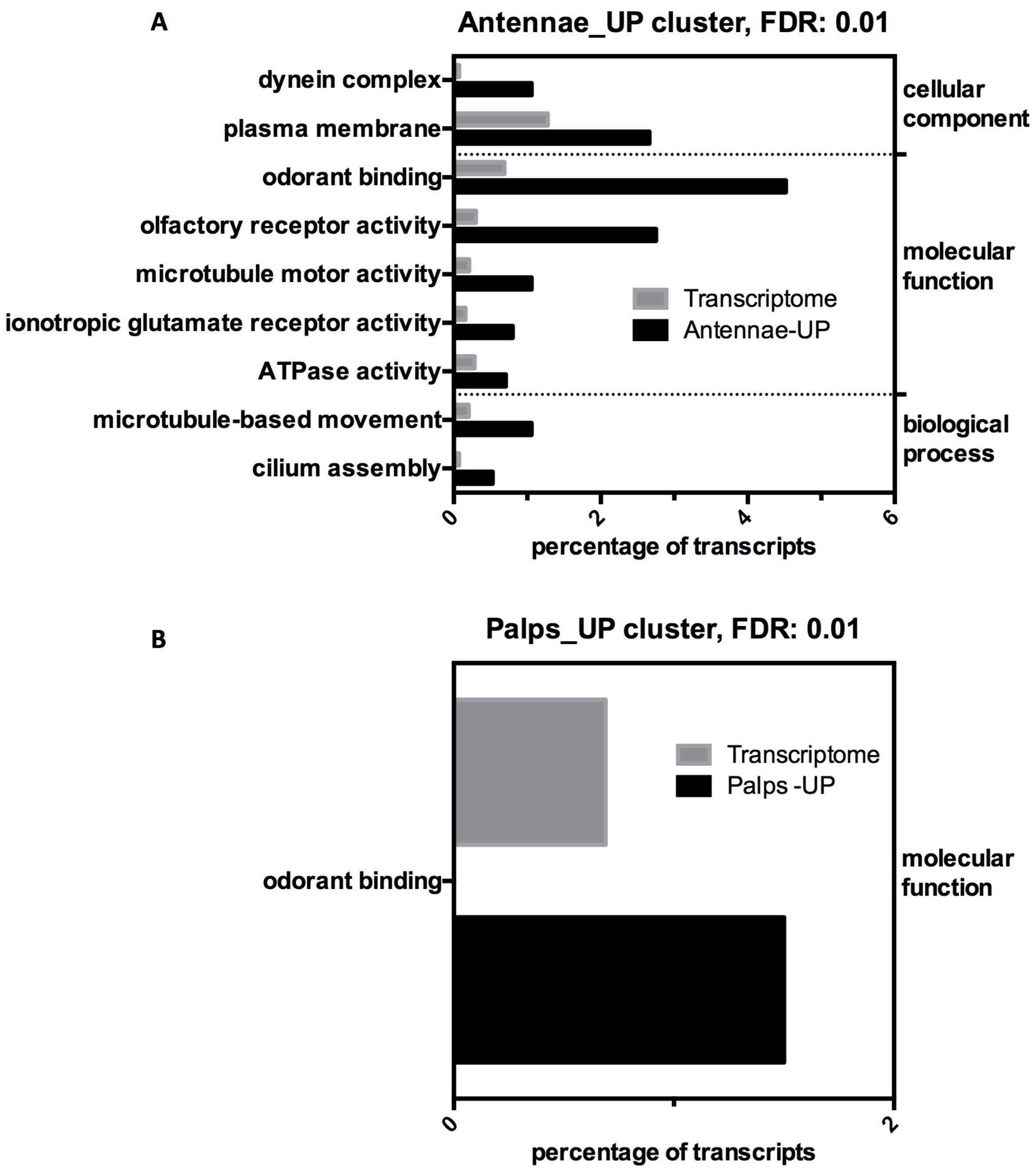

Figure S2. GO terms enrichment. GO terms frequency in antennae (A) and palps (B) specific subsets (FDR < 0.01, see Table 2) was compared to GO terms frequency of the transcriptome. GO categories significantly enriched in the antennae (A) and palps (B) subsets are shown.
