# Supplementary material for: Deciphering the olfactory repertoire of the tiger mosquito Aedes albopictus
Source: BMC Genomics. 2017 Oct 11;18:770. doi: 10.1186/s12864-017-4144-1 (PMC5637092; doi:10.1186/s12864-017-4144-1)
Supplement: Supplementary file 10 — Chemosensory proteins in the different samples and average TPM. (PDF 41 kb) [file 12864_2017_4144_MOESM10_ESM.pdf]

Table S1. Chemosensory proteins in the different samples and average TPM values.

Gene family	Transcriptome	TPM	Antennae	Palps	Male Heads	Female
**OBP**	**77**	>3	38 (2918.38)	39 (1085.10)	34 (93.90)	36 (43.64)
		>1	40 (2772.56)	43 (984.36)	39 (82.07)	40 (39.46)
		>0	70 (1584.41)	60 (705.56)	60 (53.43)	66 (23.99)
**OR**	**82**	>3	48 (38.28)	9 (84.00)	7 (37.04)	7 (49.08)
		>1	64 (29.28)	12 (63.37)	10 (26.33)	10 (34.96)
		>0	81 (23.19)	51 (15.12)	70 (3.93)	65 (5.55)
**IR**	**60**	>3	26 (16.99)	6 (21.65)	4 (22.88)	8 (8.61)
		>1	32 (14.18)	11 (12.63)	11 (9.45)	11 (6.88)
		>0	56 (8.16)	40 (3.65)	52 (2.20)	52 (1.60)
**GR**	**30**	>3	3 (50.36)	6 (162.86)	2 (41.70)	3 (23.60)
		>1	12 (13.82)	8 (122.62)	4 (21.63)	8 (10.19)
		>0	27 (6.35)	17 (57.85)	27 (3.40)	30 (2.93)

Table S1. Chemosensory proteins in the different samples and average TPM values.
Number of chemosensory genes (OBP, OR, IR and GR) in the four transcriptomes found at different abundance thresholds (TPM>0, TPM>1 and TPM>3). In brackets, average TPM corresponding to each sub-group are indicated.
