# Supplementary material for: Deciphering the olfactory repertoire of the tiger mosquito Aedes albopictus
Source: BMC Genomics. 2017 Oct 11;18:770. doi: 10.1186/s12864-017-4144-1 (PMC5637092; doi:10.1186/s12864-017-4144-1)
Supplement: Supplementary file 12 — Alignment of PlusC OBPs. (PDF 4328 kb) [file 12864_2017_4144_MOESM12_ESM.pdf]

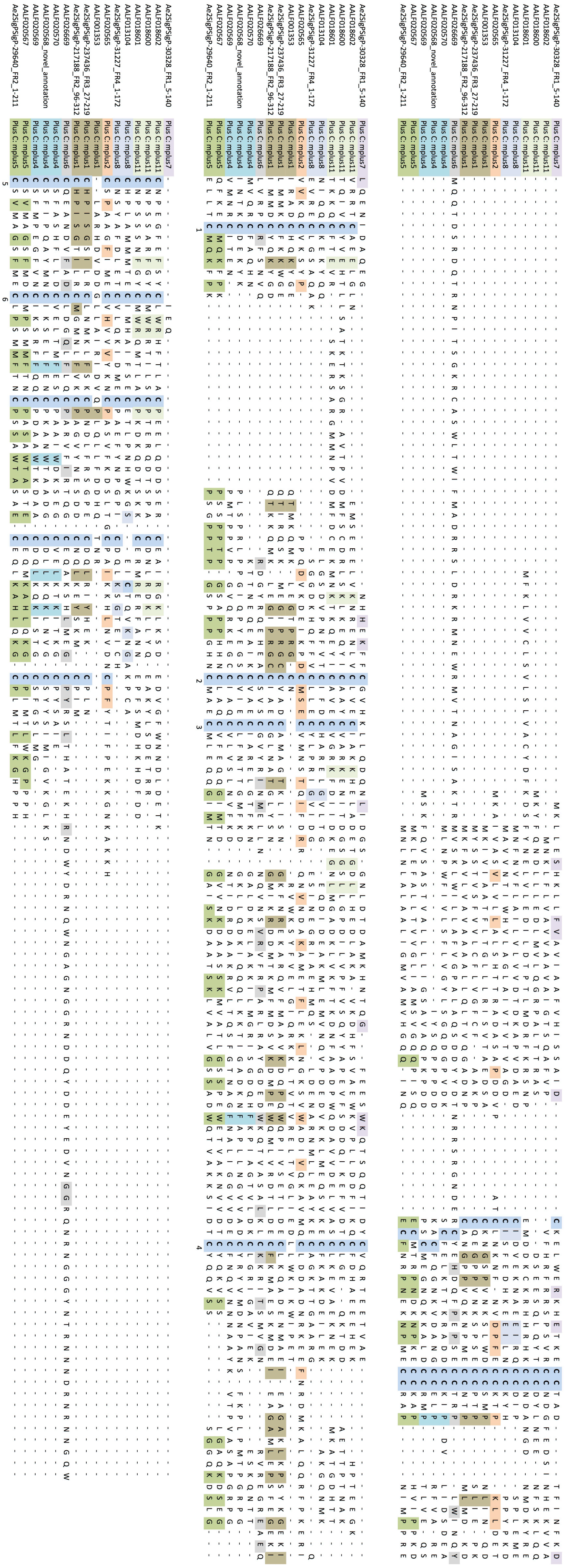

Figure S4. Alignment of PlusC OBPs. Clustal Omega alignment of the 16 PlusC OBPs identified in the *Ae. albopictus* transcriptome: sub-families or clusters as identified in Manoharan et al. (2013) are indicated on the left side. Conserved Cysteins are in bold and highlighted in light blue with the numbering reported below. Selected signatures typical of each cluster/subfamily are shown with distinctive colors.
