# Supplementary figures and images for: Deciphering the olfactory repertoire of the tiger mosquito Aedes albopictus
Source: BMC Genomics. 2017 Oct 11;18:770. doi: 10.1186/s12864-017-4144-1 (PMC5637092; doi:10.1186/s12864-017-4144-1)

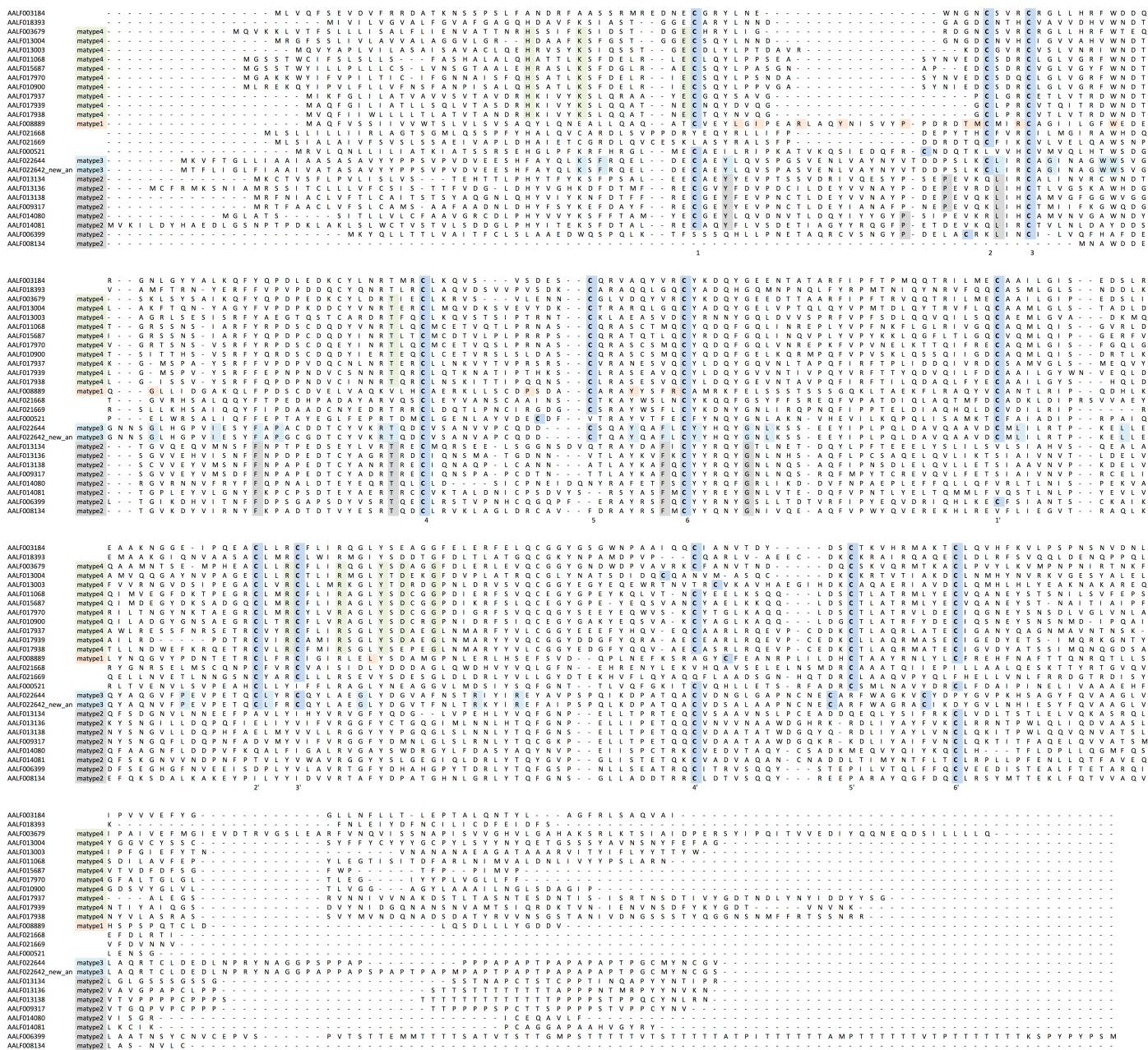

Supplement: Supplementary file 13 — Alignment of Atypical OBPs. (PDF 8211 kb) [file 12864_2017_4144_MOESM13_ESM.pdf]
