# Supplementary material for: Deciphering the olfactory repertoire of the tiger mosquito Aedes albopictus
Source: BMC Genomics. 2017 Oct 11;18:770. doi: 10.1186/s12864-017-4144-1 (PMC5637092; doi:10.1186/s12864-017-4144-1)
Supplement: Supplementary file 14 — Phylogram of OBP family members in Ae. albopictus and Ae. aegypti. (PDF 125 kb) [file 12864_2017_4144_MOESM14_ESM.pdf]

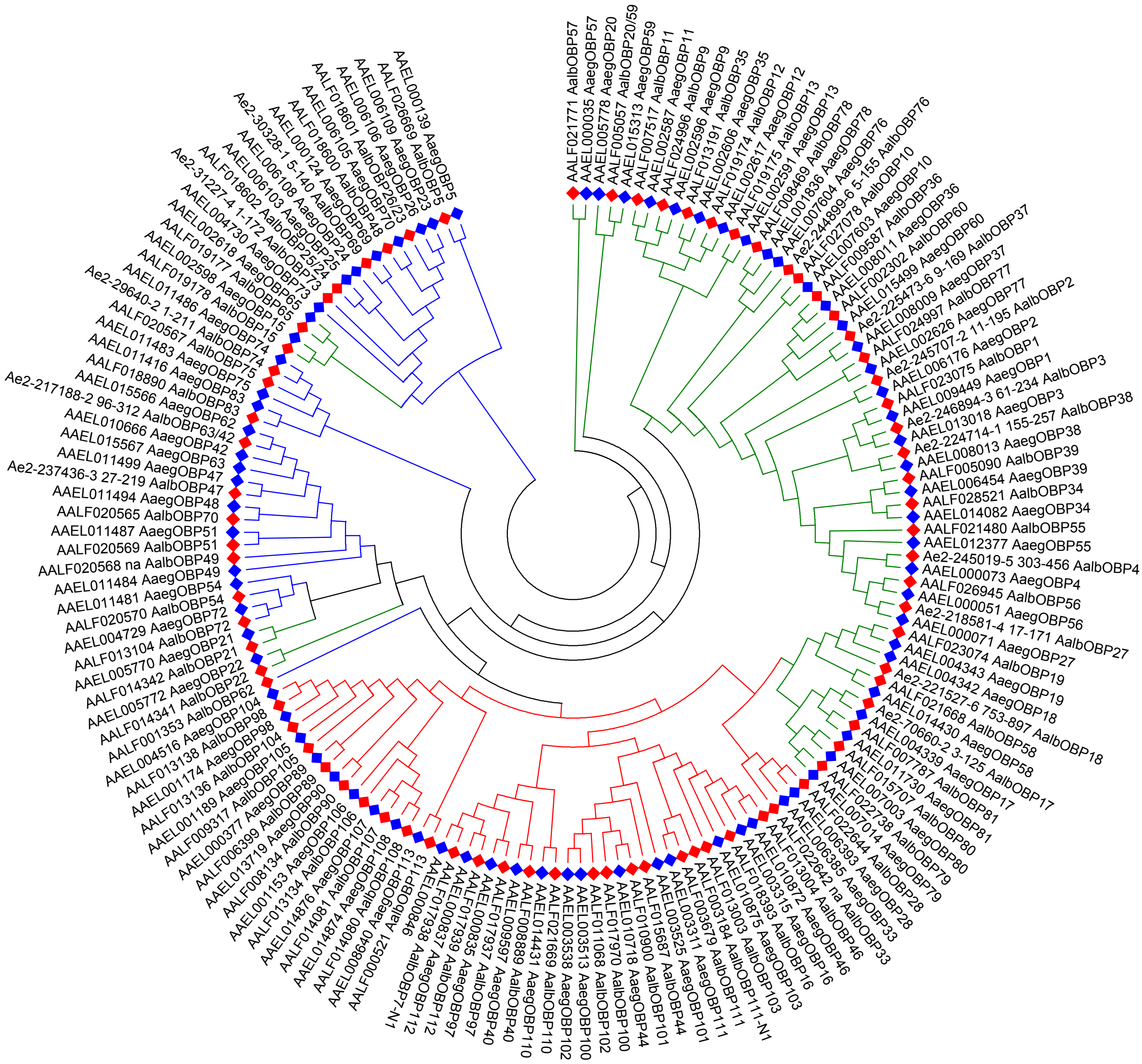

Figure S6. Phylogram of OBP family members in *Ae. albopictus* and *Ae. aegypti*. Clades or branches associated to different OBP subfamilies are shown in colors: Classic, green; PlusC, blue; Atypical, red. Red diamonds: *Ae. albopictus* ortholog; blue diamonds: *Ae. aegypti* orthologs.
