# Supplementary material for: Deciphering the olfactory repertoire of the tiger mosquito Aedes albopictus
Source: BMC Genomics. 2017 Oct 11;18:770. doi: 10.1186/s12864-017-4144-1 (PMC5637092; doi:10.1186/s12864-017-4144-1)
Supplement: Supplementary file 18 — Alignment and phylogenetic tree of OR61. (PDF 1577 kb) [file 12864_2017_4144_MOESM18_ESM.pdf]

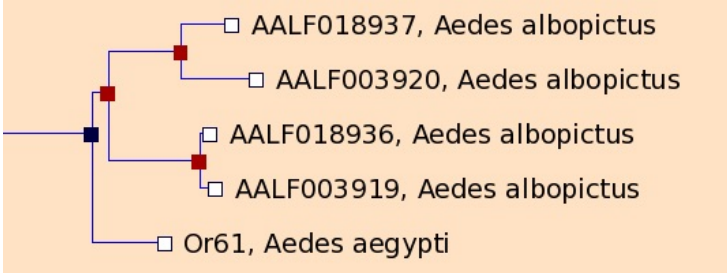


```
CLUSTAL O(1.2.3) multiple sequence alignment


AALF018937      -------------------------------------------------------MLIVM
AALF003920      ---------------------------------------------MLRCATNTNHR---I
AAEL017277      MFRSTRRRFTNWISNKTKLKPDTDVFFLLDYFLVLSGVQLLTKNGYLRTAWNVYRTLLGV
AALF003919      MFCSIKTRFTEWWKHLTKLKPGRDSFFLLDYFLVLAGIHLLTRNGYLRTVWNFYRALLFI
AALF018936      ------------------------------------------------------------

AALF018937      QVILITRKIWSIFQTEDNFQLISNSLHMCVGFSIIFTRIFFIVRSFKQ------------
AALF003920      GTVIDDRHEARALTRAD-------------GTAATSSERVSAVRSNRESSSERRRRAVAI
AAEL017277      HVLLMSRKIWSVFQTEDNFQLIANNMLMCVGCLIIFARCIFIVRSSEQ------------
AALF003919      HVVLMTRKVMEIFQTEDNFQLIANNMIMCVGCFIIFARCVFIVTSFKQ------------
AALF018936      ------------------------------------------------------------

AALF018937      ---VNGV-RCFVNQRKFRADDPAAFAIRQRTYANTINITIAMILNCFVQTVIVLFTDLAN
AALF003920      IIIIDTRRPAVNVMRKFRADDPAAFAIRQRTYANTINITIAMILNCFVQTVIVLFTDLAN
AAEL017277      ---IKIV-RRFVNERKFKADDSFAMTVRQNTYNNTIIVTVVMILNCVVQSGMILFTELGE
AALF003919      ---VNSI-RNLVNRREFQANDPTAMAIRQSAFNNAIFVTLFMAMNSLTQTFMVLFTDLGK
AALF018936      ------------------------------------------------------------

AALF018937      SESLQLPFYHRRTD----------------------------------------------
AALF003920      SESLQLPFYLTGVSSLENNLLEKIHSGMFSVYVYFASTNFLAVFLPLSTLKAEMEVVGDA
AAEL017277      AESLLLPFYLEGLSSSENRIIQKLYSGMFSVYASYASTNFLAVYLPMSTLKVELKVVVDA
AALF003919      TESLLLPFDLVGLSSSENFILQRIYSGMFSIYVFLASTNFLAVYLPLTALKAEMRIVVYS
AALF018936      ------------------------------------------------------------

AALF018937      ------------------------------------------------------------
AALF003920      FDKIMDHVDVRMAALELNEIDEIREEQFWKILQEELTQCFIAHTAVLDKVKDLKKLTDPT
AAEL017277      FEKIVHRVDERTVGKNMQPLEDLNEAVFWEILRDELTQCIRAHGAVLARVKALKKLTDPT
AALF003919      YSKITNQVEDRMSGLGHDAVDDSNEAHFWEILQDELTQCVRAHAAVLDKVGEFKKLSDPT
AALF018936      -----------MSGLGHKAVDDSNEAHFWEILQDELTQCVRAHAAVLDKVGELKKLSDPT

AALF018937      ------VTVVIQKLGVMAVLFSPQFDSFNTLSLEYSFRYTLECYSFCYVVSRFNEEHNGI
AALF003920      FLMLYYMTMLFIAVGVMAVLFSPQFDSFNTLSLEYSFRYTLECYSFCYVVSRFNEEHNGI
AAEL017277      FLLLYYMTMLFIAIGVIAVLFTPKFDSFNTISLEYTFRYTMEWYVLCYLVSNFNEEHNDI
AALF003919      FLLLYYMTMLYAAIGVIAVLFTPKLNTFNTISLEYSMRYMLECFVFCHVVSNFNEQHNNI
AALF018936      FLLLYYMTMLYAAIGVIAVLFTPKLNTFNTISLEYSMRYMLECFVFCHVVSNFNEQHNNI
                      :*::   :**:****:*::::***:****::** :* : :*::**.***:**.*

AALF018937      VNKLSQFHWGVDLRYSKRFAREYKKVRSMVLMIIMQSQKSLNFSCGGLFELTMGSFTSII
AALF003920      VNKLSHFHWGVDLRYSKRFAREYKKVRSMVLMIIMQSQKSLNFSCGGLFELTMGSFTSII
AAEL017277      AKKLSQFHWGVDLRYSKRFARDYKQIRSMVLMVIMQSQKSLNFSCGGLFELTMGSFTAVI
AALF003919      VNKLSQFHWGVDLRYSKRFSREYKQIRSMVLMIIMQSQKSLNFSCGGLFELTMGSFTTIV
AALF018936      VNKLSQFHWGVDLRYSKRFSREYKQIRSMVLMIIMQSQKSLNFSCGGLFELTMGSFTTIV
                .:***:*************:*:**::******:************************:::

AALF018937      NKTYTLTTYFWNIKQRT
AALF003920      NKTYTLTTYFWNIKQRT
AAEL017277      NKTYTLTTYFWNIKQRG
AALF003919      NKTYTLTMYFWNIKKRE
AALF018936      NKTYTLTMYFWNIKKRE
                ******* ******:*
```

Figure S7. Alignment and phylogenetic tree of OR61. Upper panel: section of Gene Tree Image from VectorBase (VBGT00190000009797) extracted by Comparative Tool. Lower panel: sequences alignment of *Ae. albopictus* and *Ae. aegypti* orthologs using Clustal Omega.
