# Supplementary material for: Deciphering the olfactory repertoire of the tiger mosquito Aedes albopictus
Source: BMC Genomics. 2017 Oct 11;18:770. doi: 10.1186/s12864-017-4144-1 (PMC5637092; doi:10.1186/s12864-017-4144-1)
Supplement: Supplementary file 19 — Alignment and phylogenetic tree of IR41p1. (PDF 1841 kb) [file 12864_2017_4144_MOESM19_ESM.pdf]

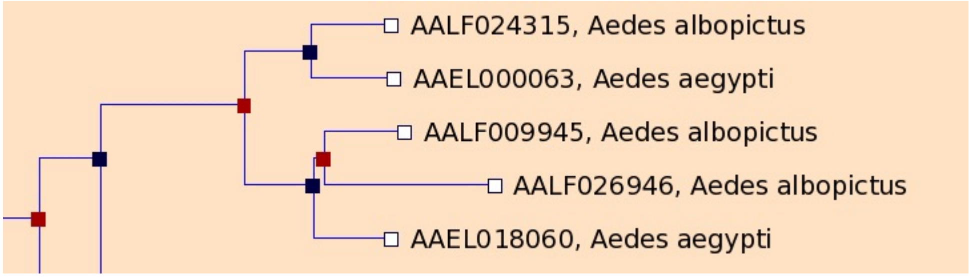


```
AAEL000063       -----------------------MEQLEPFTNITGIYTVCLIIGNLIHHLPISEILSGIP
AALF024315       ------------------------------------------------------------
AALF026946       MNRIVEYNEQSLTAVALWTSYSNILKDLLLPSIAGIYSVCVILGNLTSNFTLMKSFSNVP
AAEL018060       ----MEHTEREMKSVQHWNSYGDILSNILIPDIAGIYTVCVVFRKLVTNFSYVKTFLNVP
AALF009945       ------------------------------------------------------------

AAEL000063       YIVIRLEDIGQSNSTHNTLLTSIESGCNTFIVQHYVVLDFLNIFLEIHDQANYRNPNKYV
AALF024315       ------------------MLAGIESGCNTFIVHHHVILDFLDIFLEAHDLASYRNPNKYV
AALF026946       YEVIELDGVGQLDSTTSLMLDGIESGCNTFIIHHHVILDFLDVFVDIHDQANYRNPNKYV
AAEL018060       YVIIELEDIGQHNSTASSMLAGIESGCNSFLVQQHAVLDFLDIYTKIHDEAGYRNPNKYL
AALF009945       ------------------------------------------------------------

AAEL000063       ILLMEDHGSEGLLDSIRKHPSIWEIMNLLILLPSDERPLVELLSHRFVDTKKGFEWNLVD
AALF024315       VLLLEDHASNDLLDSIRNHPCLWEIMNFLVCQPARGGSVIELLSHRFIDYGKGFAWHLVD
AALF026946       LFLMEEPASDDFLESIRAHSSEIG------------------------------------
AAEL018060       MFLMEDASSANLLESIRGHSSMQEIMNLLIFQPSNDPSSIDLITHRYIATEKGIEWYLVD
AALF009945       ------------------------------------------------------------

AAEL000063       RYHTQRRSFEVGTELFPDKTLNLRGRPIRLATFNINPHIFLSSDDARNTEIRLGNQSYSM
AALF024315       RYHTEEHAFDFGNVLFPDRTLNLLGRSIRVATFNVNPHIFLSMDDAGNSRVKLENHSYLL
AALF026946       --------------------------------------------------LVNGDQVYSV
AAEL018060       RFHIGTSSFEMGNQLFPDKTFNLMGKPIRVATFNINPHIFLIESEIGTPVVISGNESYSL
AALF009945       ----------MGHQLFPDKTLDWMGKAIRVATFDINPHIFMVEPEIETPVIKYDNQAYSL
                                                                   :   :. * :

AAEL000063       DGLDGILLVDFCRRRNCTVDLVVDSKNMWGVIHPNRTGNGIIGNLVERRADIGLGAISSW
AALF024315       DGLNGFLLVEFCRRRNCTIDLVVDSKNMWGVIQPNRTGNGIIGNLVERRADLGIGALSNW
AALF026946       NGPNGFLLVEFCKKRNCSIELVVDSVHMWGEIYPNRTGNGILGNVVERRADFGIGAISSW
AAEL018060       NGPNGILLMEFCRRRNCTIDLVVDSVNMWGVINPNRTGNGILGNVVERRADLGIGALSSW
AALF009945       NGPNGILLMEFARRHNCSIELIV--------------GNGILGNVAERRADVGVGALSSW
                 :* :*:**::*.:::**:::*:*              ****:**:.*****.*:**:*.*

AAEL000063       YHCYSFLTFSSPDQRGGVTCLTPKPNLLPRWKIVAMVFLTSVYLIIFGTFMCSVIIYTII
AALF024315       HHCYSFLTFSAPVQRGGVTCLTPKPNLLPRWKVIAMVFLTSVYLSIIGTFVCSVAIYTTI
AALF026946       YHCFEYLSFSFAFQKGGVTCLVPKPKQLPRWKEIAMAFSTYVYLITIVSFCFVVAIYMLI
AAEL018060       YHCYQYLAFSFAYERGGVTCVVPKPKQLPRWKEVAMGFTTSVYMTIFVTFCCTVVVYMLI
AALF009945       YHCYQYLAFSFAYERGGVACLVPKPKQLPRWKEIAMGFTRSVYVAIFVAFCCIVACYILI
                 :**:.:*:**   ::***:*:.***: ***** :** *   **:  : :*   *  *  *

AAEL000063       FQCSRKKPLENSVVWNAFNVFAVFLLQSTNNIRNRSVSETVLSVALLLFSLNIAAIYSGK
AALF024315       FRYSMKNPLTKSIVWNALNVLAVFLLQSTSNFRNRSVSETIFSVALLLFSLNIAAIYSGK
AALF026946       VRFSVRSRFEKSIVWNALNVLAIYLLQSTSVVRNRSVSESLLSVTVLIFSLNLATIYSGK
AAEL018060       VRLTIQEPLERTFAWNVLNVLAIYLLQATNIVRSRSVSETLLSVAVLIFSMNLATIYLGK
AALF009945       VRFAIQGPMERSVVWHALNVLAIYLLQSTSIVRNRSASESLLSVAVLFFSMNLATIYLGK
                 .: : :  : .:..*:.:**:*::***:*. .*.**.**:::**::*:**:*:*:** **

AAEL000063       YASLRTIPLYEKEVNSLEDLAESGIPWLQAHEAWTYSLLLSKNPAVMKLVSHFKVYPAPK
AALF024315       YASLRTIPLYESAINTLEDLAKSGIPWLQAHEAWSYSLLLSKNPTIMKLVSLFKVYPIPK
AALF026946       YASLRTIPMYEPAIDSKDDLAKSGIPWVQIHEAWAYDFSMSENPLC--------------
AAEL018060       YASLRTIPMYEPAIDTKEELAKSGITWMQAHEAWSYSLRLSDNPTEVSLCSNFKVDVPPM
AALF009945       YASLRTIPMFEQAIDTKESLAQSGIPWIQAHEAWSYSLRLSKNPTEVNLLSNFRVYPLPT
                 ********::*  ::: :.**:*** *:* ****:*.: :*.**

AAEL000063       LHQIADQGGSAFAMGRLHNGHLMLGDWINSGNIHNYQLMRGDLYYEHEVAMATKTWPLIE
AALF024315       LHQIADQGGSAFAMGRLSNGHLMLGDWINAGNIHRYQLMRDDLYYEHEIAMATKTWPLIE
AALF026946       --------DAAFASARMHNGHLMLGEWIDAGNIHKYQLMKNDLYYVLKVAMSTKTWPLLE
AAEL018060       LAQIADQGNSAFALARLHNGHLMLGDWINAGNIHKYQLMQNDLYYEHEVAMATKTWPLME
AALF009945       LRQMADEGNSAFALARLHNGHLMLGDWINADNIRKYHLMRNDLYFEHEVAMATKTWPLME
                         .:*** .*: *******:**::.**:.*:**:.***:  ::**:******:*

AAEL000063       KFNELLHLTASGLLLRVQELRIMYQHNDYYVQTGALSSHEREPYQPEALAVEDVFGGLIV
AALF024315       KFNELLHQTASGLLLRPQELRIMYQHNDYYVQTVGLNSHVRAAYEPRALALEDVFGGLIV
AALF026946       RFNDLLSRTAEGLLLMVHERKNIYRYNDYYVQVVALNSHIRGENKPTALAIEDVLGCCDN
AAEL018060       RFNELLGQASDASLLLLQEYQNIFQYNDYYVQMVALNSHVRQPNEPKPLAMEDVMGGFII
AALF009945       RFNELLGQATEASLLLLKEYQNINQYNDYYVQMVALNSHIREPNEPKALAVEDVLGGLIV
                 :**:**  ::.. **  :* : : ::******  .*.** *   :*  **:***:*

AAEL000063       LGCGSLCAGVTFVIEVWLYNSQKQVSTTN--------
AALF024315       LGFGLLCAGVAFVTEVWVGKSPTVDPNDENSEKYLYE
AALF026946       REGGGVCNTVHN-------------------------
AAEL018060       LGSGLLCASVNFMIEVWILCPRKTKQVNDVE------
AALF009945       LGFGLLCAFVNFIVEIWVLCPSKTDLVKDNE------
                    * :*  *
```

Figure S8. Alignment and phylogenetic tree of IR41p1. Upper panel: section of Gene Tree Image from VectorBase (VBGT00190000009824) extracted by Comparative Tool. Lower panel: sequences alignment of *Ae. albopictus* and *Ae. aegypti* orthologs using Clustal Omega.
