# Supplementary material for: Deciphering the olfactory repertoire of the tiger mosquito Aedes albopictus
Source: BMC Genomics. 2017 Oct 11;18:770. doi: 10.1186/s12864-017-4144-1 (PMC5637092; doi:10.1186/s12864-017-4144-1)
Supplement: Supplementary file 20 — IR111.2 gene family in Ae. albopictus. (PDF 144 kb) [file 12864_2017_4144_MOESM20_ESM.pdf]

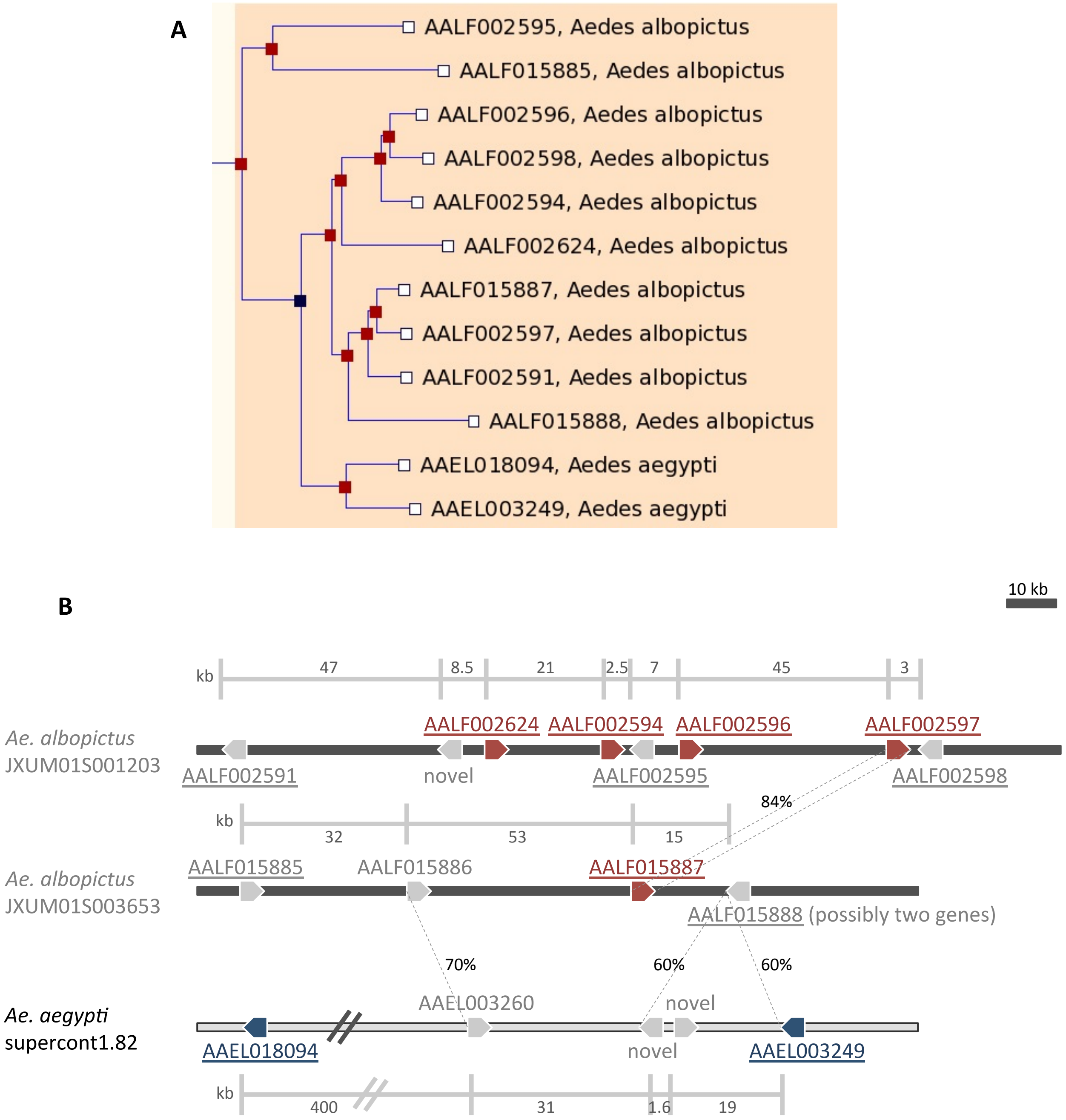


**Figure S9. IR111.2 gene family in *Ae. albopictus*.** (A) Section of Gene Tree Image from VectorBase (VBGT00730000019944) showing the ten *Ae. albopictus* homologs of *the Ae. aegypti* AaegIR111.2/ AAEL003249 and AAEL018094. (B) Schematic representation of IR111.2 gene clusters in *Ae. albopictus* and *Ae. aegypti*. Genes encoding for members of IR111.2 family are schematically placed in two and one scaffolds of *Ae. albopictus* and *Ae. aegypti*, respectively. The *Ae. aegypti* AaegIR111.2/AAEL003249 and AAEL018094 are marked in blue and the five *Ae. albopictus* homologs found in our transcriptome in red. Other annotated and putative novel family members are shown in light grey. Underlined VectorBase IDs refer to sequences found in panel A. Dotted lines indicate high percentage of sequence identity. Grey bars and numbers indicate the distance among genes (in kbp). Genes were manually annotated using the Artemis software.
