# Supplementary material for: Deciphering the olfactory repertoire of the tiger mosquito Aedes albopictus
Source: BMC Genomics. 2017 Oct 11;18:770. doi: 10.1186/s12864-017-4144-1 (PMC5637092; doi:10.1186/s12864-017-4144-1)
Supplement: Supplementary file 21 — IR7 genetic cluster. (PDF 250 kb) [file 12864_2017_4144_MOESM21_ESM.pdf]

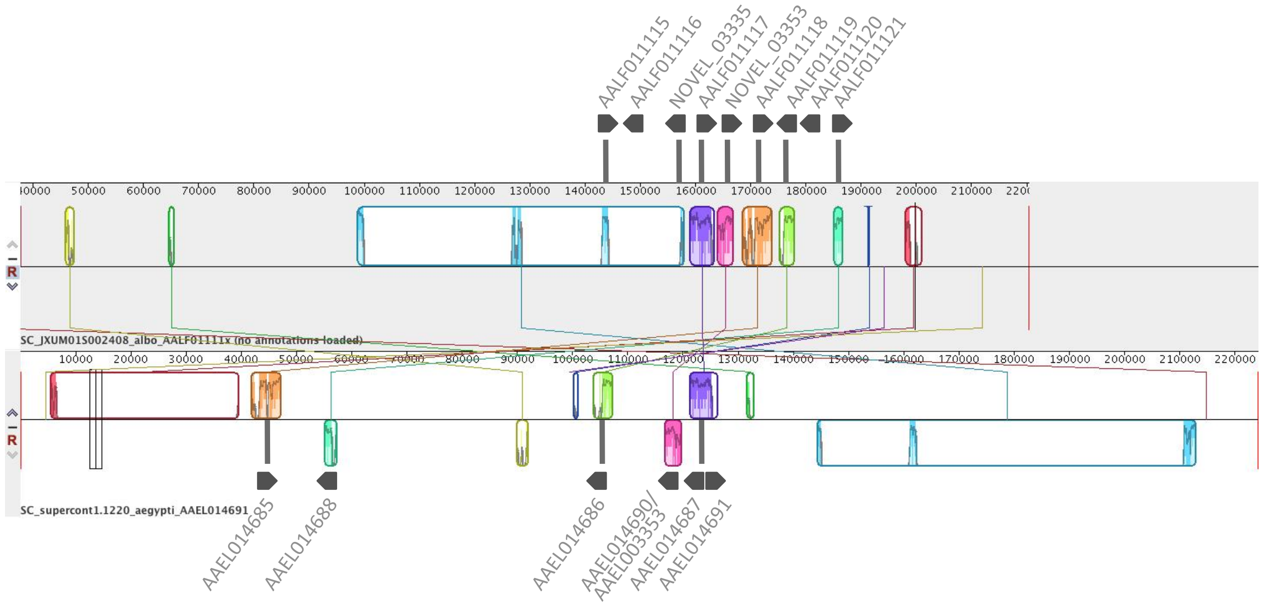

Figure S10. IR7 genetic cluster. Scaffolds (indicated in figure) containing the IR7 cluster in *Ae. albopictus* and *Ae. aegypti* were aligned using Mauve software and regions of homology highlighted with different colors. When possible (according to scaffold coordinates), genes belonging to the IR7 clusters were associated to the regions of significant homology.
