# Supplementary material for: Deciphering the olfactory repertoire of the tiger mosquito Aedes albopictus
Source: BMC Genomics. 2017 Oct 11;18:770. doi: 10.1186/s12864-017-4144-1 (PMC5637092; doi:10.1186/s12864-017-4144-1)
Supplement: Supplementary file 22 — Percentage of identity of gustatory receptors in Ae. albopictus sensory organs. (PDF 43 kb) [file 12864_2017_4144_MOESM22_ESM.pdf]

Table S4. Percentage of identity of gustatory receptors in *Ae. albopictus* sensory organs.

		**AalbGR63**	**AalbGR35**	**AalbGR20**	**AalbGR58**	**AalbGR3**	**AalbGR1**	**AalbGR2**
AALF021200	**AalbGR63**	100.00	5.88	19.35	6.82	14.80	13.14	13.64
Ae2-91626_FR6_1-238	**AalbGR35**	5.88	100.00	28.57	20.45	18.05	17.65	18.52
AALF009050	**AalbGR20**	19.35	28.57	100.00	27.61	16.79	13.97	15.94
Ae2-643_FR3_1-225	**AalbGR58**	6.82	20.45	27.61	100.00	16.39	12.73	17.74
AALF019265	**AalbGR3**	14.80	18.05	16.79	16.39	100.00	22.65	27.61
AALF013834	**AalbGR1**	13.14	17.65	13.97	12.73	22.65	100.00	36.92
AALF019900	**AalbGR2**	13.64	18.52	15.94	17.74	27.61	36.92	100.00

Table S4. Percentage of identity of gustatory receptors in *Ae. albopictus* sensory organs. “Percent Identity Matrix” as created by Clustal2.1 (Clustal Omega at http://www.ebi.ac.uk/Tools/msa/clustalo/). ID are reported in the first column.
